# Conformational diversity facilitates antibody mutation trajectories and discrimination between foreign and self-antigens

**DOI:** 10.1073/pnas.2005102117

**Published:** 2020-08-27

**Authors:** Deborah L. Burnett, Peter Schofield, David B. Langley, Jennifer Jackson, Katherine Bourne, Emily Wilson, Benjamin T. Porebski, Ashley M. Buckle, Robert Brink, Christopher C. Goodnow, Daniel Christ

**Affiliations:** ^a^Garvan Institute of Medical Research, University of New South Wales (UNSW) Sydney, Darlinghurst, NSW 2010, Australia;; ^b^St Vincent's Clinical School, Department of Medicine, UNSW Sydney, NSW 2052, Australia;; ^c^Department of Biochemistry and Molecular Biology, Biomedicine Discovery Institute, Monash University, Clayton, VIC 3168, Australia;; ^d^Medical Research Council Laboratory of Molecular Biology, CB2 OQH Cambridge, United Kingdom;; ^e^UNSW Cellular Genomics Futures Institute, UNSW Sydney, NSW 2052, Australia

**Keywords:** clonal selection, humoral immunity, somatic hypermutation, autoantibody redemption, affinity maturation

## Abstract

Conformational diversity and self-cross-reactivity of antigens have been correlated with evasion from neutralizing antibody responses. We utilized single cell B cell sequencing, biolayer interferometry and X-ray crystallography to trace mutation selection pathways where the antibody response must resolve cross-reactivity between foreign and self-proteins bearing near-identical contact surfaces, but differing in conformational flexibility. Recurring antibody mutation trajectories mediate long-range rearrangements of framework (FW) and complementarity determining regions (CDRs) that increase binding site conformational diversity. These antibody mutations decrease affinity for self-antigen 19-fold and increase foreign affinity 67-fold, to yield a more than 1,250-fold increase in binding discrimination. These results demonstrate how conformational diversity in antigen and antibody does not act as a barrier, as previously suggested, but rather facilitates high affinity and high discrimination between foreign and self.

## Significance

Conformational diversity of foreign antigens and cross-reactivity with self are implicated in the failure to generate effective antibody responses against many challenging pathogens, but few studies directly address how these two factors affect antibody formation. Here we address this question from biophysical, structural, and immunological perspectives using structurally related lysozyme proteins. The results show germinal centers have remarkable ability to select antibody producing cells along novel hypermutation trajectories, transmuting an antibody with no capacity to differentiate foreign from self into highly foreign-specific antibody derivatives, exploiting conformational flexibility in antigen and antibody. These findings address a central issue for developing vaccines against HIV and other chronic infections and represent a prime example of stepwise, evolutionary adaptation of protein–protein interfaces.

Antibodies display exquisite specificity to distinguish between macromolecules, largely preventing autoantibody production in response to pathogens that mimic self (1). While monospecificity is often sought for diagnostic and therapeutic uses, broader reactivity is preferred when mounting broadly neutralizing antibodies (bnAbs) against viruses that rapidly mutate, exemplified by the HIV Env protein and the influenza virus hemagglutinin protein. These rare bnAbs are only observed in a small subset of individuals, often after several years of infection; such antibodies tend to be characterized by noncanonical structures, high mutational loads in antibody variable genes, conformational diversity (2–5), and polyspecificity for many other antigens including binding to self-antigens (6–9).

Broader reactivity is also preferred in the preimmune antibody repertoire, where most nascent antibodies are self-reactive (10, 11). Germline antibodies, and some affinity-matured antibodies, adopt alternative conformations enabling binding of different antigens (12–16). This may increase the probability of initiating antibody responses against foreign antigens, but requires balance against self-reactivity and the probability that B cell development is inhibited by immune tolerance checkpoints (17).

Conformational diversity of foreign antigens is an equally important and poorly understood variable. Important vaccine targets are often disordered or surrounded by flexible loops such as the circumsporozoite protein (CSP) (18) and merozoite surface protein 2 (MSP2) (19), the gp41 and gp120 trimer of HIV targeted by the self-reactive 2F5 bnAb (20–23), and the stem region of influenza hemagglutinin (24). Glycans on viral envelope proteins are conformationally flexible and many viral proteins are cloaked by self-glycans. Literature on the immune properties of disordered antigens is highly conflicted. On one hand, flexible regions of *Staphylococcus* *aureus* fibronectin binding protein are proposed to prevent immune recognition (25). The disordered V3 loops of HIV gp120 are thought to mask critical neutralization sites (21, 26). Rigidifying the HIV Env V2 loop increased its recognition by germline precursors (27). Similarly, epitopes with reduced conformational disorder in MSP2 were more antigenic than flexible epitopes (28). Conversely, Ofek et al. demonstrated that flexibility of gp41 epitope scaffolds correlated positively with immunogenicity (22), and similar conclusions have been reached from studies of anti-peptide antibodies (29). It has been proposed that disordered antigens result in high-specificity but low-affinity antibodies, resulting from the entropic cost associated with disorder–order transitions (26, 30). Complicating affinity, epitope disorder could also alter the rate constants for macromolecular associations (31, 32). Another theory proposes that flexible epitopes may in fact be immunodominant but detrimental to immunity, functioning as a “smokescreen,” distracting the immune system from critical targets (33), and that cross-reactive, flexible epitopes on *Plasmodium* *falciparum* interfere with normal affinity maturation (34). There are numerous examples of productive antibodies targeting disordered epitopes in pathogen protection including those to MSP2 (19) and CSP (18, 35, 36). Furthermore, examination of the immune epitope database suggested that disordered epitopes were equally selected (37) and that antibodies to disordered epitopes had similar numbers of somatic hypermutations, suggesting that the entropic cost may not affect affinity maturation (37). However, none of these studies examine the role of conformational diversity in antibody discrimination between similar foreign and self-antigens.

To understand the conformational basis for selection or counterselection of cross-reactivity against foreign and self-antigens during antibody hypermutation in germinal centers, we used a mouse model where the cross-reacting foreign and self-antigens are well-defined lysozyme proteins (38). Here we examined whether a foreign lysozyme with only one contact residue difference from self, but with altered conformational flexibility, could be immunologically discriminated, and what antibody mutation trajectories were employed. We reveal mutation trajectories employing both CDR and framework (FW) mutations that present a vastly altered paratope to complement the distinctly different conformation presented by the flexible antigen. These results provide structural insights into the somatic evolution of antibody specificity for foreign versus self-antigens, and the role of conformational flexibility in antigen and antibody.

## Results

### Generation of an Antigen Variant with Increased Conformational Flexibility.

**Fig. 1.**
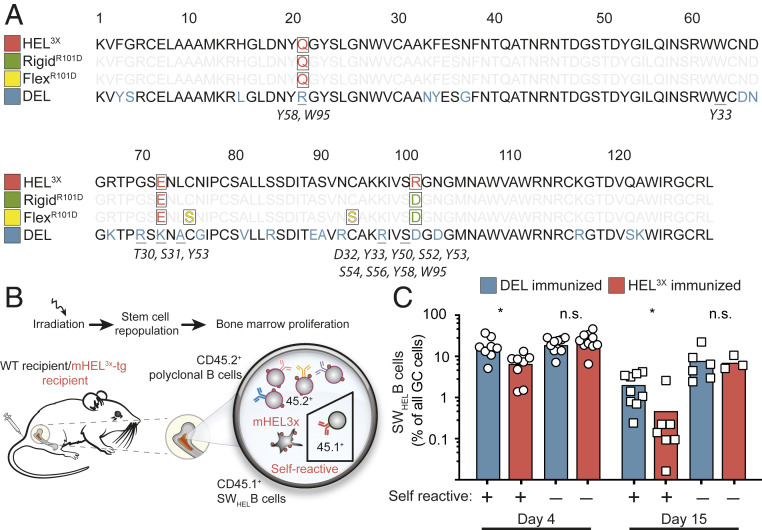
Autoantibody redemption upon immunization with self and foreign antigens. (*A*) HEL^3X^ self-lysozyme, Rigid^R101D^, Flex^R101D^, and DEL antigen sequences. Positions highlighted in red differ from parental HEL lysozyme. Underlined positions contact heavy chain (antibody residues listed in italics). (*B*) Generation of hematopoietic chimeras with a minority of Hy10-expressing SW_HEL_ B cells (CD45.1^+^) developing among polyclonal CD45.2^+^ B cells in transgenic mice ubiquitously expressing HEL^3X^ self-lysozyme in a membrane-bound form (mHEL^3X^). For comparison, control chimeras comprised WT mice lacking mHEL^3X^ self-antigen. (*C*) Percentage of CD45.1^+^ SW_HEL_ cells among GC B cells in mHEL^3X^ self-lysozyme transgenic (self-reactive: +) or WT (self-reactive: −) recipient mice immunized with DEL or HEL^3X^ antigen coupled to SRBCs. Each data point represents one animal. Data are pooled from two experiments per time point with three to four mice per group. *P < 0.05, n.s. = not significant; Student’s *t* test.

To investigate the impact of conformational flexibility of foreign antigen on antibodies that cross-react with self, we analyzed mice to track and sequence HyHEL10 (Hy10)-derived antibodies on single B cells responding to four structurally similar lysozyme proteins (Fig. 1*A*). “Self” lysozyme protein (mHEL^3X^) (38, 39) was expressed on the surface of all mouse cells under control of the ubiquitin gene promoter. Duck egg lysozyme (DEL) differs from self-lysozyme at 25 amino acids, including five directly contacting the Hy10 antibody (Fig. 1*A*). By contrast, Rigid^R101D^ lysozyme was identical to self-lysozyme except for one arginine-to-aspartate charge reversal at residue 101, at the contact surface with Hy10 antibody. Flex^R101D^ lysozyme also displayed this single contact difference from self-lysozyme but also carried the mutations Cys76Ser and Cys94Ser, which bridge the lysozyme α-helical and β-sheet domains and comprise one of four disulfide bonds that make the lysozyme fold conformationally robust (*SI Appendix*, Figs. S1*B* and S4) (40). The Hy10 antibody does not contact either cysteine but binds to surface residues on the lysozyme α-helical and β-sheet domains and binds with comparable low–intermediate affinity to self-lysozyme, Flex^R101D^, and DEL (1/*K*_D_ = 1.1 × 10^7^ M^−1^, 1.6 × 10^7^ M^−1^, and 2.5 × 10^7^ M^−1^, respectively *SI Appendix*, Fig. S2 *A*–*C*).

The lysozyme fold remains relatively stable when Cys76 and Cys94 are mutated to alanine or serine, as analyzed by a range of biophysical techniques, including nuclear magnetic resonance (NMR) (41, 42), circular dichroism (43, 44), and crystallography. Crystallographic data for the analogous human C77A lysozyme where this disulfide bond is disrupted show two conformers—one effectively wild type (WT) (Protein Data Bank [PDB] entries 1LZ4 and 1LHM; albeit with elevated temperature factors indicative of local instability) (45, 46), and the other with residues 73 to 78 adopting an alternative “swung-out” conformation (PDB entries 207L and 208L, in part due to derivatization of the unpaired cysteine) (47), suggesting that flexibility between these extremes might be possible in solution, as confirmed by a local absence of local nuclear Overhauser effects for this region when analyzed by NMR (48) and supported by molecular dynamics (MD) simulations (*SI Appendix*, Fig. S1).

### Clonal Redemption of Autoreactive B Cells Requires Differences between Self- and Foreign Antigens.

**Fig. 2.**
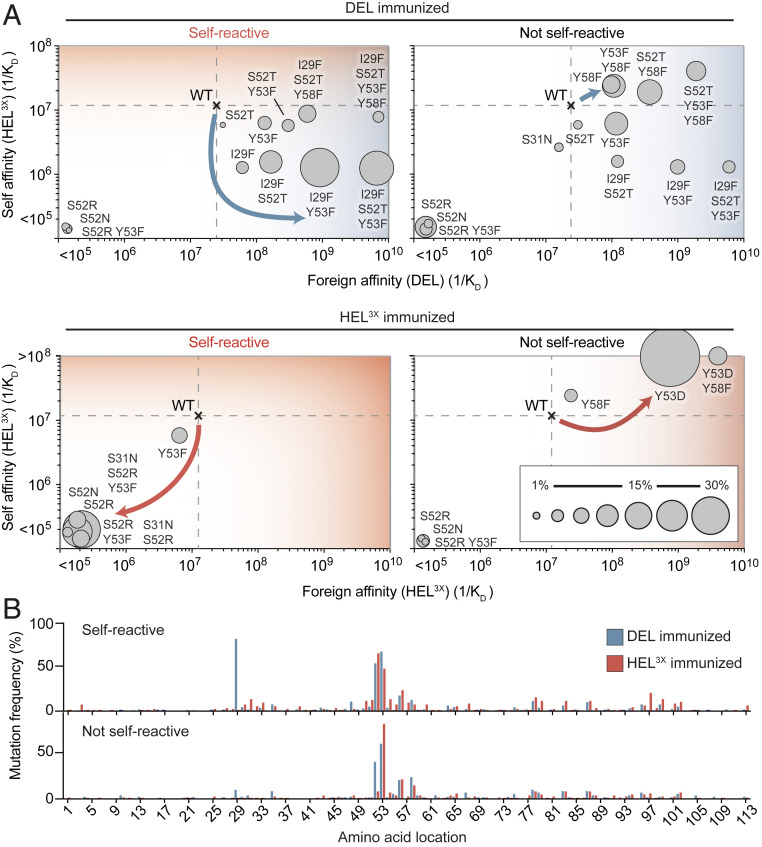
Autoantibody redemption requires a path for escape of self-reactivity. (*A*) Mutational trajectories of Hy10-expressing SW_HEL_ B cells upon immunization with foreign or self-antigen (day 15). CD45.1^+^ GC B cells were subjected to single-cell sequencing. Dashed lines show the affinities of the WT founder antibody for self and foreign proteins. Circles show the affinities of recurring mutant antibodies for self and foreign proteins. Areas of the circles denote the percentages of CD45.1^+^ GC B cells with the indicated mutations. Arrows indicate predominant mutational trajectories in the presence (*Left*) or absence (*Right*) of membrane-bound HEL^3X^ self-lysozyme. (*B*) Percentage of CD45.1^+^ GC cells with substitutions at antibody variable heavy-chain positions. Data are pooled from two experiments per time point with three to four mice per group.

To track selection of B cells bearing self-reactive antibodies we engineered bone marrow chimeric mice ubiquitously expressing self-lysozyme on cell surfaces as previously described (38) (Fig. 1*B*), in which the majority of mature B cells were polyclonal, carrying the CD45.2^+^ marker but ∼0.1% of mature follicular B cells were CD45.1^+^ SW_HEL_ immunoglobulin knockin B cells expressing the Hy10 antibody. When the mice were immunized with DEL coupled to sheep red blood cells (SRBCs), many self-reactive Hy10 B cells accumulated in germinal centers (GCs) (Fig. 1*C*) and acquired antibody V_H_-domain mutations I29F, S52T, and Y53F that specifically lowered affinity to self-lysozyme while improving binding to foreign DEL (Fig. 2*A*) (38). To further investigate this phenomenon, we solved the X-ray crystal structure of self-lysozyme (HEL^3X^), enabling direct comparison to the structure of DEL (PDB entry 5v8g) (*SI Appendix*, Fig. S1*A* and Table S1). Superposition of the structures revealed the backbone of the self and foreign antigens to be extremely similar (rmsd of 0.29 Å for 94 Cα atoms) with differences primarily restricted to surface side chains. Despite multiple surface differences between the lysozymes, a surface pocket unique to the foreign DEL, created by the substitution of Leu75 to alanine (*SI Appendix*, Fig. S1*A*), proved to be the main difference exploited by Hy10 cells mutating away from self-binding (38).

By contrast with DEL immunization, when parallel sets of mice expressing self-lysozyme were immunized with HEL^3X^ self-lysozyme coupled to SRBCs, Hy10 B cells tended to be less frequent in the GC compared to DEL-SRBC immunized mice, despite other GC B cells being comparable in number (Fig. 1*C* and *SI Appendix*, Fig. S3 *G* and *H*). In response to self-lysozyme-SRBC a very different antibody V_H_-domain mutation, S52R, was carried by 60% of Hy10 GC cells, including 30% where S52R was paired with Y53F (Fig. 2 *A* and *B* and *SI Appendix*, Fig. S3*M*). Bio-layer interferometry revealed that the S52R mutation, alone or in combination with the Y53F mutation, abolished measurable binding to the highest concentration of self-lysozyme tested, representing at least 100-fold lower affinity (*SI Appendix*, Table S2). Thus, when foreign SRBCs displayed a protein structurally identical to self, the Hy10 trajectory adopted was mutation away from self-recognition.

To confirm the impact of self-lysozyme on the mutation trajectories, parallel sets of bone marrow chimeric mice lacking expression of self-lysozyme were immunized with DEL-SRBC or HEL^3X^ nonself-lysozyme-SRBC. When the Hy10-expressing B cells were not confronted with the self-reactivity conundrum, the I29F mutation trajectory elicited by DEL-SRBC was uncommon and the main mutation trajectory was to accumulate high frequencies of GC cells with V_H_ mutations which increase affinity for DEL and self-lysozyme (Fig. 2 *A* and *B* and *SI Appendix*, Fig. S3*M*) (38, 39, 49). Following HEL^3X^ self-lysozyme-SRBC without self-reactivity, few Hy10 GC cells followed the affinity-lowering S52R mutation trajectory. Instead almost all had acquired V_H_ mutations Y53D (84% of cells), Y58F (10%), or both, that increase affinity for HEL^3X^ (self-lysozyme). Analysis of the serum IgG_1_ compartment revealed that the presence of self-lysozyme on the mouse cells diminished IgG_1_ antibody titers 10-fold when mice were immunized with HEL^3X^ self-lysozyme-SRBC (*SI Appendix*, Fig. S3 *B*, *E*, and *F*). Thus, cross-reactivity between a foreign antigen and a ubiquitous self-antigen dooms GC B cell selection to a loss-of-binding trajectory when foreign and self-epitopes are identical, but redirects to alternative pathways yielding foreign-specific high-affinity antibodies when topological differences can be exploited between foreign and self-epitopes.

### Antigen Flexibility Allows Resolution of Foreign/Self-Reactivity by Enabling New Evolutionary Trajectories and Increased Mutational Diversity.

**Fig. 3.**
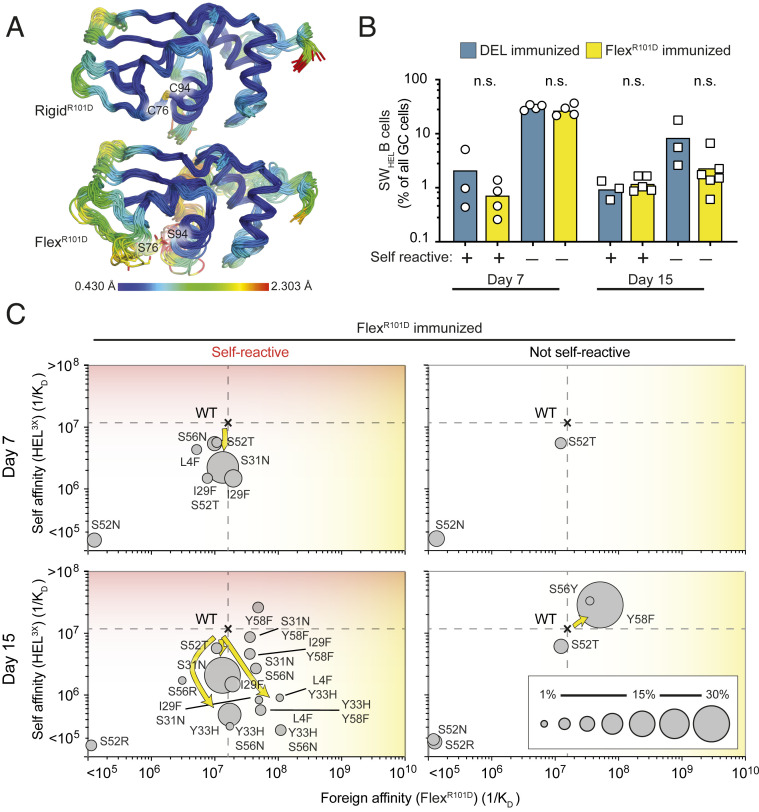
Autoantibody redemption against a flexible antigen. (*A*) MD simulations. Cartoons colored by root mean squared fluctuation of each residue, averaged across three independent simulations. Flex ^R101D^ remains overall stable throughout the simulation, with increased local flexibility observed among residues 73 to 78. (*B*) Percentage of CD45.1^+^ cells among GC B cells. mHEL^3X^ self-lysozyme (+) or WT (−) recipient chimeras were immunized with DEL or Flex^R101D^ coupled to SRBCs. Each data point represents one animal. (*C*) Mutational trajectories of Hy10-expressing B cells upon immunization with flexible antigen. CD45.1^+^ GC B cells were subjected to single-cell sequencing. Dashed lines show the affinities of WT founder antibody for self and foreign proteins. Circles show the affinities of recurring mutant antibodies for self and foreign proteins. Areas of the circles denote the percentages of CD45.1^+^ GC B cells with the indicated mutations. Arrows indicate predominant mutational trajectories in the presence (*Left*) or absence (*Right*) of membrane-bound HEL^3X^ self-lysozyme. Data are representative of three independent experiments with one to two mice per group. n.s. = not significant; Student’s *t* test.

While DEL presents a rigid epitope with multiple surface differences from self-lysozyme, we next examined mutation trajectories in response to Flex^R101D^. The antigens are characterized by equivalent Hy10 affinities (1/*K*_D_ = 2.5 × 10^7^ M^−1^ and 1.6 × 10^7^ M^−1^, respectively *SI Appendix*, Fig. S2); however, in marked contrast to DEL, Flex^R101D^ presents only a single surface difference from HEL^3X^ self but in the context of a more flexible epitope (Figs. 1*A* and 3*A*). Immunization with Flex^R101D^-SRBC 7 or 15 d prior to B cell harvest resulted in similar accumulation of Hy10-expressing CD45.1^+^ B cells in the GC as DEL immunization, regardless of whether self-lysozyme was present or absent on the surface of the mouse’s own cells (Fig. 3*B* and *SI Appendix*, Fig. S4 *B* and *C*). Compared to the dominant I29F trajectory elicited within 7 d after DEL immunization, Flex^R101D^ predominantly induced new and unique mutation trajectories with equal rapidity, comprising V_H_ substitutions L4F, S31N, Y33H, and S56N that also decreased affinity for self-lysozyme 3- to 39-fold while preserving affinity for foreign Flex^R101D^ (Fig. 3*C* and *SI Appendix*, Figs. S4*J* and S5 and Table S2). These foreign self-discriminating mutations were not selected when self-lysozyme was absent on the mouse’s cells: instead more slowly arising Y58F or S56Y mutations modestly increased affinity for both Flex^R101D^ and self-lysozyme (Fig. 3*C* and *SI Appendix*, Table S2).

**Fig. 4.**
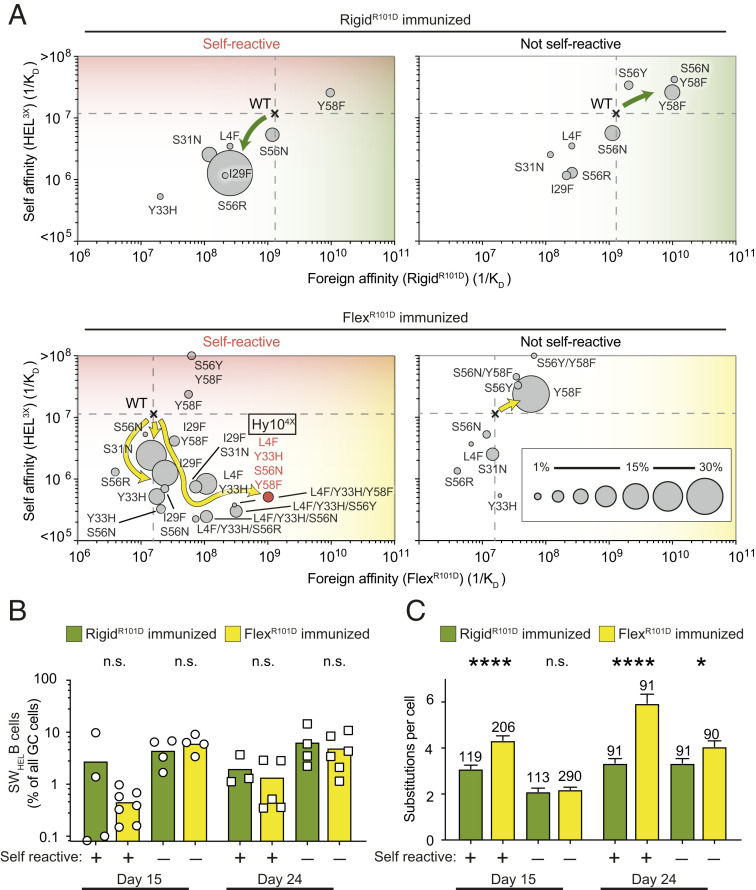
Antigen flexibility enables B cells to explore diverse mutational trajectories leading to loss of self-reactivity and high affinity for foreign antigen. (*A*) Mutational trajectories of Hy10-expressing B cells upon immunization with Rigid^R101D^ or Flex^R101D^ antigen coupled to SRBCs (day 24). CD45.1^+^ GC B cells were subjected to single-cell sequencing. Dashed lines show the affinities of WT founder antibody for self and foreign proteins. Circles show the affinities of recurring mutant antibodies for self and foreign proteins. Areas of the circles denote the percentages of CD45.1^+^ GC B cells with the indicated mutations. Arrows indicate predominant mutational trajectories in the presence (*Left*) or absence (*Right*) of membrane-bound HEL^3X^ self-lysozyme. High-affinity Hy10^4X^ quadruple mutant (Flex^R101D^ immunization) highlighted in red. (*B*) Percentage of CD45.1^+^ cells among GC B cells. mHEL^3X^ self-lysozyme (+) or WT (−) recipient mice were immunized with Rigid^R101D^ or Flex^R101D.^. Data points represent one chimera. (*C*) Average number of substitutions per CD45.1^+^ GC B cell. *P < 0.05, ****P < 0.0001, n.s. = not significant; Student’s *t* test. Data are pooled from two independent experiments with two to three mice per group.

The effect of epitope flexibility was analyzed further by comparing GC mutation trajectories in mice immunized 15 to 24 d earlier with Flex^R101D^ or Rigid^R101D^, the latter presenting an identical single surface difference but with the epitope rigidified by an intact Cys76-Cys94 disulfide bond. By 24 d after immunization of self-lysozyme-expressing mice, Flex^R101D^ elicited GC B cells making antibodies with various combinations of L4F, Y33H, S56N, and Y58F mutations that decrease affinity for self 20-fold to 1/*K*_D_ = 2.7 × 10^5^ M^−1^, while increasing foreign affinity to 10^8^ to 10^9^ M^−1^ (Fig. 4*A* and *SI Appendix*, Figs. S2, S6*H*, and S7 and Table S2). The greatest discrimination between self and foreign was achieved by antibodies combining four mutations (Hy10^4X^)—L4F, Y33H, S56N, and Y58F—yielding 1/*K*_D_ = 5.4 × 10^5^ M^−1^ against self and 1.04 × 10^9^ M^−1^ affinity against foreign, representing a 1,900-fold difference (*SI Appendix*, Table S2). Then next best were antibodies having three of these mutations—L4F, Y33H, and S56N—yielding a 400-fold differential between 1/*K*_D_ = 2.7 × 10^5^ M^−1^ against self and 1.09 × 10^8^ M^−1^ affinity against foreign.

These mutations were rare or undetectable in mice immunized with Rigid^R101D^, and instead the predominant trajectory acquired a single S56R mutation that decreased affinity for self-lysozyme from 1/*K*_D_ = 1.1 × 10^7^ M^−1^ to 1.24 × 10^6^ M^−1^, but also decreased affinity for Rigid^R101D^ from 1/*K*_D_ = 1.36 × 10^9^ M^−1^ to 2.7 × 10^8^ M^−1^, representing a 5-fold decrease in foreign binding (Fig. 4*A* and *SI Appendix*, Fig. S2 and Table S2) therefore only representing an ∼200-fold discrimination between self and foreign. Mutation away from self-binding was more efficient in mice immunized with the flexible foreign epitope: 33.9% of Hy10-expressing GC B cells had lowered their affinity below 1/*K*_D_ = 10^6^ M^−1^, primarily representing cells with Y33H, whereas only 0.8% of cells had achieved this in mice immunized with the rigid epitope (Fig. 4*A*). Commensurately, Hy10 on GC B cells induced by Flex^R101D^ carried a much higher number of amino acid substitutions than GC B cells induced by Rigid^R101D^ (Fig. 4*C* and *SI Appendix*, Fig. S6*G*). Few Hy10-expressing GC cells followed these affinity-lowering trajectories in mice lacking self-lysozyme expressed on their own cells, and the Hy10 cells accumulated to higher frequencies as a percentage of all GC and memory B cells when they did not need to resolve self-reactivity from foreign reactivity (Fig. 4*A*). Similarly, self-reactive mice immunized with the flexible epitope had 10-fold lower self-binding IgG_1_ than those immunized with the rigid epitope (*SI Appendix*, Fig. S6 *I* and *J*).

### Long-Range Structural Rearrangements of Antibody Framework and CDR Regions Enable High-Affinity Capture of Flexible Antigen.

**Fig. 5.**
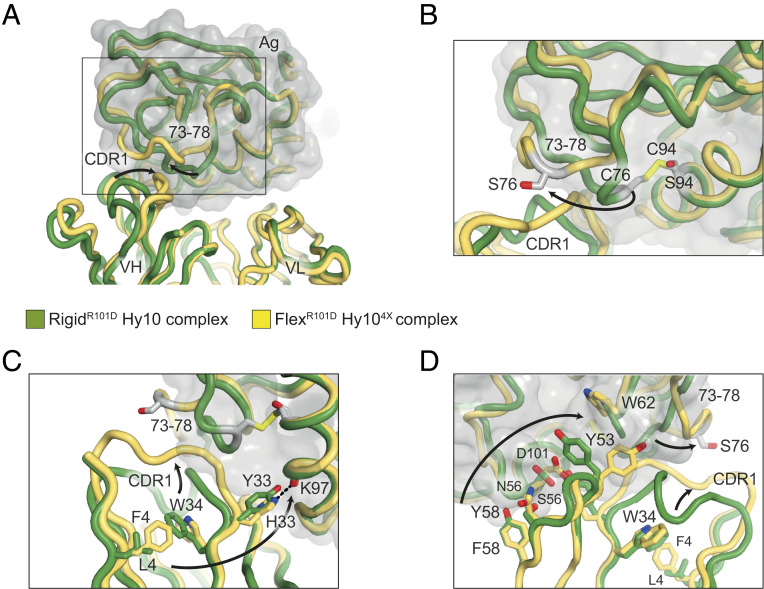
Structural basis of autoantibody redemption against a flexible antigen. (*A*) Structures of Rigid^R101D^ (in green) and Flex^R101D^ (in yellow) antigens were determined in complex with Hy10 and Hy10^4X^ (with V_H_ mutations L4F, Y33H, S56N, and Y58F) antibodies, respectively (PDB identifiers 6p4d and 6p4A). The structures superpose with high similarity with the exception of residues 73 to 78 of the antigens and the CDRH1 antibody region. (*B*) Conformational change in Flex^R101D^ (arrow) enabled by disulfide bond disruption from the C76S and C94S mutations. The altered antigen conformation is stabilized by the rearrangement of the CDRH1 region of the Hy10^4X^ antibody variable heavy domain (in yellow). (*C*) Structural rearrangement of the CDRH1 region (upper arrow) mediated by long-range rearrangement of a network of antibody framework residues (lower arrow). The spatial linkage of LF4 and Y33H antibody mutations, observed early in the Flex^R101D^ mutational trajectory, is mediated through a conserved tryptophan W34 antibody framework residue. The larger F4 side chain repositions the W34 side chain (as well as of I29 and adjacent residues; *SI Appendix*, Fig. S8*E*); this rearrangement is stabilized by the Y33H imidazole side chain forming new hydrogen bonds (dashed line and *SI Appendix*, Fig. S8*A*). (*D*) Alternative perspective of CDRH1 framework and antigen rearrangements (right arrows). Hy10^4X^ antibody mutations Y58F and S56N involve a domino-like rearrangement of side chains (left arrow): local structural adjustments through antigen D101, antibody Y53, and the indole side chain of antigen W62, result in large-scale rearrangement of antigen positions 73 to 78 and antibody CDRH1 (right arrows).

Given the unique L4F, Y33H, S56N, and Y58F mutation trajectories induced by Flex^R101D^, we next used crystallography to understand the structural basis for how these four mutations combined to convert an antibody with nearly identical affinity for self and foreign into one with 1,900-fold self-foreign discrimination. We solved the structure of the quadruply mutated Hy10 (Hy10^4X^) alone and in complex with Flex^R101D^ in a Fab format (*SI Appendix*, Table S1). We also solved the structure of the complex between Rigid^R101D^ and Hy10 Fab, thus allowing structural comparison of both flexible and rigid antigen–antibody structures of comparable affinities (1/*K*_D_ = 1.04 × 10^9^ M^−1^ and 1.36 × 10^9^ M^−1^, respectively). When structurally aligned, the overall Flex^R101D^ and Rigid^R101D^ complexes superpose tightly (antigen-Fv components with rmsd of 0.49 Å over 258 Cα positions), with localized differences observed in the antibody–antigen interfaces (Fig. 5). The primary difference between the Flex^R101D^ and Rigid^R101D^ complexes involves an antigen surface loop, residues 73 to 78, that in the Flex^R101D^ structure adopts a swung-out conformation enabled by the lack of disulfide tether normally covalently linking residues C76 and C94 (Fig. 5*B*) similar to a human C77A lysozyme variant where the same disulfide bond is missing (45, 46). The swung-out flex conformation (the loop in question untethered by contacts with symmetry-related molecules) is further enabled and stabilized by altered conformations within the antibody component of the structure, whereby mutations at both framework and hypervariable positions result in CDRH1 adopting a different conformation that complements the altered antigen structure (Fig. 5 and *SI Appendix*, Fig. S8).

Enabling this antibody conformational change is an ensemble of Hy10 mutations: L4F, Y33H, S56N, and Y58F. L4F and Y33H, which emerge early in the trajectory and often in combination, are connected *in trans* via a conserved framework residue, W34, which rearranges its large indole side chain to sterically accommodate the leucine-to-phenylalanine change at position 4 (Fig. 5*C*). The flipped indole, in turn, would ordinarily sterically clash with the side chain of I29, which forms part of the Hy10^4X^ CDRH1 region displaced to the new conformation (*SI Appendix*, Fig. S8*C*). The Y33H mutation results in one hydrogen bond being lost but two hydrogen bonds being forged by its bidentate imidazole side chain: one with antigen (at position K97) (Fig. 5*C*) and the other to the peptide backbone at antibody heavy chain position 53 (*SI Appendix*, Fig. S8). In addition to main chain interactions, Y53 also adopts a new side chain conformation in the Hy10^4X^-Flex^R101D^ complex, forming part of a set of interface residues (involving both antibody heavy chain and antigen) that alter their conformations in a “domino-like” rearrangement (Fig. 5*D*): heavy chain Y58F mutation subtly readjusts the neighboring S56N, with the larger asparagine side chain contacting a rearranged D101 antigen residue, whose side chain inhabits space formerly occupied by Y53. The new conformation of Y53 is accommodated by a subtle shift of the side chain conformation of antigen position W62, and through the 73- to 78-loop rearrangement (Fig. 5*D*). Additional hydrogen bonds and a salt bridge further stabilize the new local conformation of Flex^R101D^ and Hy10^4X^ together (*SI Appendix*, Fig. S8).

When comparing the Hy10^4X^-Flex^R101D^ and Hy10-Rigid^R101D^ complex structures, the surface buried in the two complexes is comparable; in Flex^R101D^, antibody V_H_ and V_L_ domains bury 544 A^2^ and 362 A^2^ of antigen surface (total of 906 Å^2^), while in the rigid complex, the V_H_ and V_L_ domains bury 566 A^2^ and 325 A^2^ (total of 891 Å^2^). Comparison of the Fab components of the Hy10^4X^-Flex^R101D^ and Hy10-Rigid^R101D^ complexes to the Fab-only structure of Hy10^4X^ show that, in the absence of antigen, Hy10^4X^ CDRH1 broadly resembles the conformation observed in the rigid complex (with the exception of minor CDR adjustments to accommodate the L4F mutation and altered W34 conformation (*SI Appendix*, Fig. S8)). Taken together, the Hy10^4X^ mutations enable the antibody to adopt different conformations, retaining essentially the WT structure of the founder antibody in the absence of antigen, while allowing for structural adjustments and picomolar affinity binding in the presence of the antigen. Hence, increased flexibility within the antigen component is compensated by structural diversity within antibody framework and CDR regions.

## Discussion

Our findings provide insights into three key questions about the relationship between conformational diversity in antigen and antibody and cross-reactivity between foreign and self-antigens.

Firstly, does antibody affinity maturation derive from restriction of conformational diversity of the antibody combining site, progressing from conformationally diverse, but potentially autoreactive, germline precursors toward more specific and conformationally restricted variants (12, 14, 15)? In the response to Flex^R101D^ antigen studied here, a substantially altered antibody CDR conformation was acquired through L4F and Y33H framework mutations, similarly to what has been observed in the evolution of an HIV bnAb (2). Here, this maturation trajectory was only selected when driven by rapid selection of the L4F and Y33H mutations to lose self-reactivity and was not observed when Flex^R101D^ immunization occurred in mice lacking the cross-reacting self-antigen. These results are consistent with an emerging view that progressive binding site rigidification is not an obligate component of affinity maturation, and that gaining conformational diversity through antibody hypermutation can improve foreign specificity, breadth, and affinity (50, 51).

A second key question is: Does hypermutation-induced acquisition of antibody conformational diversity increase self-reactivity (4–9)? In the results here, the quadruple mutant antibody became dramatically more specific for foreign over self, through 19-fold loss of self-affinity and 67-fold gain of foreign affinity. It is notable that most of the mutation trajectories selected to lose self-reactivity but preserve foreign reactivity cease losing self-affinity around 1/*K*_D_ = 10^6^. This suggests that submicromolar self-affinities do not trigger the immune tolerance checkpoints in germinal center B cells. These thresholds are nevertheless likely to vary among self-antigens depending on their density on cell surfaces (mHEL^3X^ is expressed at low levels) or valency and concentration in solution, and the counteracting strength of T cell help promoting continued division of cross-reactive cells in the germinal center. Other factors that may contribute to the positive relationship between conformational diversity, specificity, and foreign affinity here are a single defined foreign and self-antigen, whereas in HIV bnAbs the antibody mutation trajectories are attempting to cover a large range of varying foreign epitopes while minimizing binding to a diverse array of structurally related self-glycoproteins and structurally unrelated self-proteins.

Finally, does conformational flexibility in the immunogen compromise affinity maturation (21, 26–28)? It is notable that on-rates were similar for binding DEL, Flex^R101D^, and Rigid^R101D^ (*SI Appendix*, Table S1). Nevertheless, antigen flexibility enabled affinity maturation by initiating a mutation trajectory for self–foreign discrimination founded on the L4F and Y33H rearrangements of the framework region. When self-antigen was not present, however, there was limited affinity maturation primarily by acquisition of Y58F which increased affinity only 3.7-fold to 1/*K*_D_ = 5.8 × 10^7^ M^−1^. This contrasts with our previous results with rigid DEL (38), where 100- to 1,000-fold affinity maturation occurs in the presence or absence of self-antigen, albeit by different mutation trajectories from those described here. Thus antigen flexibility appears here to impede affinity maturation unless a new antibody mutation trajectory is opened up by selection to lose cross-reactivity with self.

A striking result here is the diversity of antibody mutation trajectories employed within 7 to 24 d for clonal redemption of self-foreign cross-reactive antibodies on initially anergic B cells. Some trajectories, like L4F Y33H S56N Y58F, maximize foreign–self discrimination by first diminishing self-affinity and then increasing foreign affinity. Others only diminish self-affinity, exemplified here by I29F and S31N singleton mutations in CDRH1. Despite I29F lowering self-affinity 10-fold (a redeeming mutation) and appearing as a single mutation in self-reactive germinal center B cells induced by Flex^R101D^, I29F did not become prevalent or combine with other mutations to create trajectories for greater discrimination between foreign Flex^R101D^ and self. Similarly, S31N lowers self-affinity 4-fold but neither increases nor decreases foreign affinity, and was not followed by a mutation trajectory to increase foreign–self discrimination. High prevalence of S31N only in the presence of self-antigen shows that germinal center selection to lose self-binding operates independently from conventional affinity maturation and effectively even for a relatively modest decrease in self-affinity. Finally, some singleton mutations greatly decrease affinity for both foreign and self. These were only prevalent when foreign and self were indistinguishable (S52R elicited by HEL^3X^-SRBC) or foreign bound with higher affinity than self to the founder Hy10 antibody (S56R elicited by Rigid^R101D^). Like S31N, these mutations cannot be explained by conventional affinity maturation but reflect an independent process rapidly selecting cells with less self-reactivity.

The findings here extend the evidence that clonal redemption can be utilized as a potential strategy to generate antibodies against challenging antigens, including those mimicking self or utilizing epitope flexibility. We have found that GC B cells are able to concurrently overcome the challenges imposed by molecular mimicry and epitope flexibility to generate high-affinity, foreign-specific antibodies from antibody precursors that bind equally to foreign and self. However, a high mutational load including noncanonical framework region mutations are required to overcome these combined challenges. This supports previous evidence suggesting that a high frequency of germline precursors may be a limiting factor in HIV bnAb production (52) and may help to explain the highly mutated and atypical nature of such antibodies and why they are only generated in a limited number of patients (2, 3).

## Materials and Methods

### Animal Studies.

Mice used in this study were bred at the Australian BioResources and held at the Garvan Institute of Medical Research in specific pathogen-free conditions. The Garvan Animal Ethics Committee approved all mice protocols and procedures. C57BL/6 (nontransgenic) mice were purchased from the Australian BioResources (Moss Vale). Hy10-transgenic (SW_HEL_) mice have been described previously (53). These mice carry a single copy V_H_10 anti-HEL heavy chain variable region coding exon targeted to the endogenous *Igh*^*b*^ allele plus multiple copies of the V_H_10-κ anti-HEL light-chain transgene. SW_HEL_ mice on a CD45.1 congenic (*Ptprc*^*a/a*^) C57BL/6 background were also homozygous *Rag1*^*−/−*^ (26, 54), which prevented endogenous Ig variable region gene rearrangements so that all B cells expressed the Hy10 B cell receptor (BCR). HEL^3X^ self-lysozyme is an R21Q, R73E, and D101R triply mutated HEL protein (55) which binds with low–intermediate affinity (1/*K*_D_ =1.1 × 10^7^ M^−1^) to Hy10. Transgenic mice on the C57BL/6 background expressed HEL^3X^ as an integral cell surface protein, by addition at the C terminus of the transmembrane segment and cytoplasmic tail of the H2K^b^ class I major histocompatibility protein (39). The transgene was controlled by the human ubiquitin C (*UBC*) promoter, resulting in membrane-bound HEL^3X^ (mHEL^3X^) expression on the surface of all nucleated and anucleate cells.

### Bone Marrow Chimeras.

Recipient mice of 8 to 12 wk of age were lethally irradiated (2 × 425 cGy) using an X-RAD 320 Biological Irradiator (Precision X-Ray). Femoral, humeral, and tibial bone marrow cells were aspirated into RPMI (Gibco) with 10% heat-inactivated fetal calf serum (FCS) (Gibco), 2 mM l-glutamine, and 100 U/mL penicillin RPMI media (Gibco). Fifteen hours after irradiation, recipient mice were transplanted with an i.v. injection of 5 to 10 × 10^6^ bone marrow cells. For mHEL^3X^ transgenic (CD45.2^+^) recipients, injected bone marrow cells were 80% of SW_HEL_.*Rag1*^*−/−*^ (CD45.1^+^) origin and 20% of mHEL^3X^ transgenic (CD45.2^+^) origin. Control nontransgenic mice were reconstituted with less (45%) SW_HEL_.*Rag1*^*−/−*^ (CD45.1^+^) and more (55%) wild-type (CD45.2^+^) bone marrow, so that comparable frequencies of anti-HEL B cells in the transitional subset of the spleen were obtained in chimeras irrespective of self-antigen (mHEL^3X^) expression, as described previously (38). Chimeras were analyzed 8 to 14 wk after reconstitution.

### Protein Expression and Purification.

Purified HEL^WT^ was purchased from Sigma-Aldrich. Recombinant HEL^3X^ and DEL proteins (Fig. 1) were secreted in *Pichia pastoris* and purified from culture supernatants by ion exchange chromatography as previously described (39, 53, 55–57). The DEL used in this study is the D65N + N103D double mutant of the DEL-II isoform used previously in this model (38, 56). The amino acid sequences corresponding to the HEL or Hy10 Fab heavy- and light-chain variants were cloned into the pCEP4 expression vector (Thermo Fisher Scientific) for transient expression in Expi293 cells (Thermo Fisher Scientific), essentially as previously described (58). Fab heavy chains contained a C-terminal his-tag for purification purposes. Transformations were performed as per the manufacturer’s instructions. In the case of Fab expression, equal equivalents of heavy- and light-chain plasmid were used. Due to limited yields when expressed in isolation, for structural studies Flex^R101D^ was coexpressed and purified in complex with Hy10^4X^ Fab. For this purpose, a 1:1:1 ratio of antigen, heavy- and light-chain plasmid was used in transformations. HEL^3X^ and Rigid^R101D^ were purified from cell culture supernatant via affinity chromatography employing immobilized anti-lysozyme VHH camelid D2L19 (59). Hy10 variants or the complex with Flex^R101D^ were purified from cell culture supernatant using Talon resin (Takara). Fractions were further purified by gel filtration using a S200 26/60 column plumbed with 25 mM Tris (pH 8.0), 150 mM NaCl as the running buffer. The Rigid^R101D^-Hy10 complex was formed prior to gel filtration by combining Rigid^R101D^ and Hy10 at a 3:1 ratio. Finally, peak fractions were pooled and concentrated with spin filters (Amicon Ultracel 10KMWCO, Merck).

### Structural Studies.

Crystals of HEL^3X^ spontaneously grew at a concentration of 12 mg/mL in a solution comprising gel-filtration buffer; 25 mM Tris (pH 8.0), 150 mM NaCl. Crystals were suspended for 10 s in mother liquor supplemented with 25% (vol/vol) glycerol, prior to being looped and flash frozen in liquid nitrogen. Crystals of Rigid^R101D^-Hy10 were grown in a vapor-diffusion hanging-drop format by combining equal volumes (2 μL) of protein complex (5.5 mg/mL) with well solution (100 mM BisTrisPropane [pH 7.0], 22% [wt/vol] PEG3350). Crystals of Flex^R101D^-Hy10^4X^ were grown in a vapor-diffusion hanging-drop format by combining equal volumes (2 μL) of protein complex (3.4 mg/mL) with well solution (100 mM citrate [pH 4.2], 1 M LiCl, 11% [wt/vol] PEG3350). Crystals of Hy10^4X^ Fab alone grew out of conditions in which Flex^R101D^ was also present. The vapor-diffusion hanging drop experiment involved combining an equal volume (400 nL) of protein complex (6.8 mg/mL) with well solution (Molecular Dynamics screen JCSG+, condition C2; 100 mM citrate [pH 4.0], 1 M LiCl, 20% [wt/vol] PEG6000). This commercial screen was dispensed with a Mosquito liquid handling robot (TTP Labtech), as employed for other crystals before optimizations were performed in 24-well plates. Apart from the HEL^3X^ crystal, no explicit cryoprotection protocol was employed, and crystals were directly flash frozen in liquid nitrogen. X-ray diffraction data were recorded at the Australian Synchrotron on beamline MX2 using a Dectris Eiger ×16M detector. In each case, a 360° sweep of data was recorded, then deconvolution into 3,600 images (0.1° each). Reflections were indexed and integrated using either iMOSFLM (HEL^3X^) (60) or XDS (Rigid^R101D^-Hy10, Flex^R101D^-Hy10^4X^, Hy10^4X^) (61). Space groups were determined with POINTLESS (62) and scaling and merging were performed with AIMLESS (63), both part of the CCP4 suite of software (64). Structures were determined by molecular replacement using PHASER (65), with the search model for the HEL^3X^ structure comprising a high-resolution HEL template (PDB entry 1lks) (66), and the search model for the other structures comprised of HEL and/or variable and constant domain pairings (domains V_H_ + V_L_, and C_H_1 + C_L_1) of the complex between HEL and Hy10 (PDB entry 3d9a) (67). Components of the 3d9a-derived search models were omitted, HEL residues 73 to 78, and Hy10 heavy-chain residues 26 to 34. Rigid body and rounds of restrained B-factor refinement were performed with REFMAC5 (68), interspersed with inspection and manual model modification using COOT (69). Waters were modeled into *f*_obs_-*f*_calc_ density providing their positions satisfied sensible H-bonding networks. Loops omitted from search models were added late in refinement. Diffraction data and model refinement statistics are shown in *SI Appendix*, Table S1.

In comparing the structures of Hy10^4X^ unliganded or bound to Flex^R101D^, and Hy10 complexed with Rigid^R101D^, we considered if different crystal contacts could affect potential flexibility. All three crystallized in different orthorhombic space groups, precluding use of isomorphism to simplify the scrutiny of differences. However, in the case of the Flex^R101D^ complex, the untethered loop (disulfide removed), is relocated to a position in space where the atoms involved make no contact with symmetry-related molecules, and adopts a swung-out position similar to a human C77A lysozyme variant where the same disulfide bond is missing (45, 46).

### SRBC Conjugation.

HEL proteins were desalted into conjugation buffer (0.35 M D-mannitol [Sigma] and 0.01 M sodium chloride [Sigma]) using PD-10 columns (Amersham) as described previously (38, 70). For conjugation, SRBCs were washed three times in phosphate-buffered saline (PBS) and once in conjugation buffer as described previously (38). SRBCs were then resuspended in a final volume of 1 mL conjugation buffer containing 10 µg/mL. The solution was mixed on a platform rocker on ice for 10 min. One hundred microliters of 100 mg/mL *N*-(3-dimethylaminopropyl)-*N*-ethylcarbodimide hydrochloride (Sigma) was then added and the solution was mixed for a further 30 min on ice. SRBCs were then washed four times in PBS. Confirmation of successful conjugation was performed by flow-cytometric analysis of SRBCs using Alexa Fluor 647-conjugated Hy9 antibody. A total of 2 × 10^8^ conjugated or unconjugated SRBCs were i.v. injected into each chimeric mouse.

### Flow Cytometry.

On the day of harvest, organs were collected into PBS containing 1% bovine serum albumin (BSA) (Bovostar), cell suspensions passed through a 70-µm cell strainer (Falcon), and centrifuged 1,500 rpm (440 × *g*) for 5 min at 4 °C. Fc receptors were blocked with unlabeled anti-CD16/32 (eBioscience) before staining. To detect HEL mutant-binding cells, cells were stained with 2 µg/mL (0.14 μM) of the respective HEL mutant protein, followed by Alexa Fluor 647-conjugated Hy9. Anti-IgG_1_-FITC (BD Pharmingen) stains were followed by 5% mouse serum before staining for other surface molecules. Cells were filtered using 35-µm filter round-bottom FACS tubes (BD Pharmingen) immediately before data acquisition on a LSR II analyzer (BD Pharmingen). Forward- and side-scatter threshold gates were applied to remove red blood cells and debris and ∼5 to 7 × 10^6^ events were collected per sample. Cytometer files were analyzed with FlowJo software (FlowJo LLC).

### Analysis of Binding Affinity.

Purified Hy10 Fab fragments were buffer exchanged into PBS using equilibrated ZebaSpin columns (Thermo Fisher Scientific). Fab samples were requantified and incubated with EZ-Link NHS-PEG4-Biotinylation reagent (Thermo Fisher Scientific) at a 5:1 biotin-to-protein ratio. Free biotin was removed from the samples by repeating the buffer exchange step in a second ZebaSpin column equilibrated with PBS. Affinity of interactions between biotinylated Fabs and purified lysozyme proteins by Biolayer Interferometry (BLItz, ForteBio), essentially as previously described (71). Streptavidin biosensors were rehydrated in PBS containing 0.1% wt/vol BSA for 1 h at room temperature. Biotinylated Fab was loaded onto the sensors “on-line” using an advanced kinetics protocol, and global fits were obtained for the binding kinetics by running associations and dissociations of lysozyme proteins at a suitable range of molar concentrations. The global dissociation constant (*K*_D_) for each 1:1 Fab-lysozyme interaction was determined using the BlitzPro 1.2.1.3 software.

### Single-Cell Sorting.

Cell suspensions were prepared and germinal center B cells identified as for flow cytometry as described previously (38, 70). Single-cell sorting into 96-well plates (Thermo Fisher Scientific) was performed on a FACSAria or FACSAriaIII (BD Pharminigen). B cells from each mouse were analyzed individually to ensure that overrepresentation of one particular clone did not affect mutation analysis. The *VDJ*_*H*_ exon of the Hy10 heavy-chain gene was amplified from genomic DNA by PCR, sequenced, and analyzed as previously described (39).

### MD Simulations.

HEL^R101D^ rigid and the HEL^R101D^ flex C76S/C94S variant were protonated according to states at pH 7.0 and placed in an explicitly solvated TIP3P water box with borders of 12 Å. Na^+^ and Cl^−^ ions were added as necessary to neutralize the total charge. This system was parameterized by the AMBER ff16SB all-atom force field. Each protein was subjected to energy minimization and equilibration before production simulations were run in the NPT ensemble. Each simulation was allowed to run for 1,000 ns and repeated independently three times using AMBER 16.

### ELISA.

ELISA detection of serum concentrations of antibodies binding to HEL mutant proteins were measured as previously described (53). High-binding plates (Corning) were coated with the respective mutant protein and bound serum antibody quantified using the same IgH chain isotype-specific secondary antibodies used for flow cytometry. Antibody levels were quantified against Hy10 standards.

### Quantification and Statistical Analysis.

GraphPad Prism 6 (GraphPad Software) was used for data analysis. When the data were normally distributed, an unpaired Student’s *t* test was performed for analysis. When data were not normally distributed, Welsh’s correction was applied. For all tests, *P* < 0.05 was considered statistically significant. Unless otherwise stated error bars represent arithmetic mean. Flow cytometric plots of multiple samples are presented as mean and SE or mean. For all figures, data points indicate individual mice. *P < 0.05, **P < 0.01, ***P < 0.001, ****P < 0.0001.

## Supplementary Material

Supplementary File

Supplementary File

Supplementary File

## Data Availability

Protein structure data have been deposited in Protein Data Bank (PDB) (entries 6p4a, 6p4b, 6p4c, and 6p4d).
