# Supplementary material for: Conformational diversity facilitates antibody mutation trajectories and discrimination between foreign and self-antigens
Source: Proc Natl Acad Sci U S A. 2020 Aug 27;117(36):22341–50. doi: 10.1073/pnas.2005102117 (PMC7486785; doi:10.1073/pnas.2005102117)
Supplement: Supplementary File [file pnas.2005102117.sapp.pdf]

PNAS
www.pnas.org

Supplementary Information for

Conformational diversity facilitates antibody mutation trajectories and discrimination between foreign and self antigens

Deborah L. Burnett†[1,2], Peter Schofield†[1,2], David B. Langley†[1], Jennifer Jackson[1], Katherine Bourne[1], Emily Wilson[3], Benjamin T. Porebski[4], Ashley M. Buckle[3], Robert Brink[1,2], Christopher C. Goodnow[1,2,5]* and Daniel Christ[1,2]*.

[1] Garvan Institute of Medical Research, UNSW Sydney, 384 Victoria Street, Darlinghurst NSW 2010, Australia
[2] UNSW Sydney, NSW, Australia.
[3] Department of Biochemistry and Molecular Biology, Biomedicine Discovery Institute, Monash University, Clayton VIC 3168, Australia
[4] Medical Research Council Laboratory of Molecular Biology, Francis Crick Avenue, Cambridge CB2 OQH, UK
[5] UNSW Cellular Genomics Futures Institute, UNSW Sydney, NSW, Australia.
† Authors contributed equally.

* Christopher C. Goodnow and Daniel Christ

Email: c.goodnow@garvan.org.au (C.C.G.); d.christ@garvan.org.au (D.C.).

This PDF file includes:

Supplementary text
Figures S1 to S7
Tables S1 to S2

Other supplementary materials for this manuscript include the following:

Movies S1

www.pnas.org/cgi/doi/10.1073/pnas.2005102117

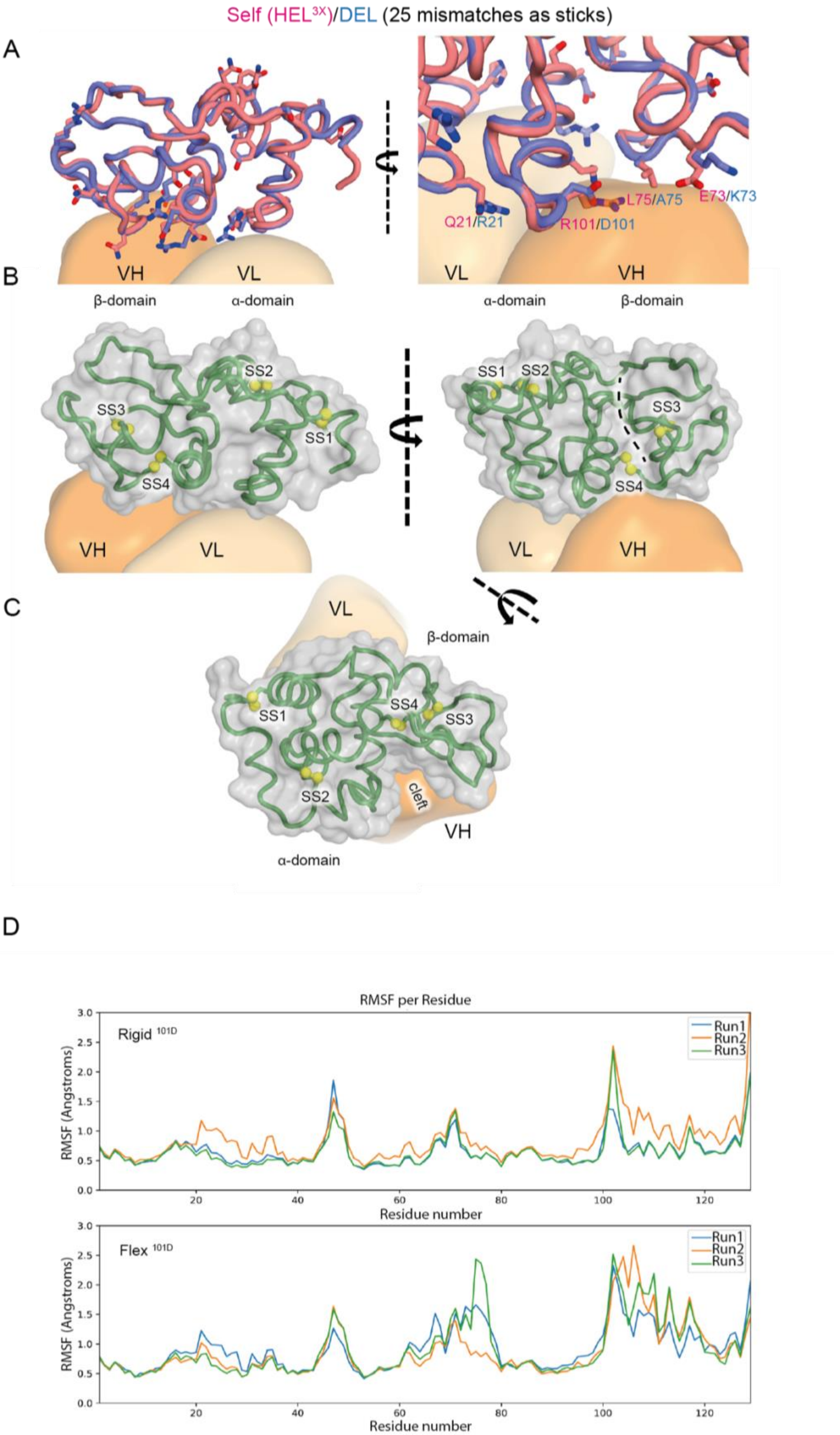
Self (HEL3X)/DEL (25 mismatches as sticks)
A
VH
VL
Q21/R21
R101/D101
L75/A75
E73/K73
VL
VH
B
β-domain
α-domain
α-domain
β-domain
SS2
SS3
SS1
SS4
SS1
SS2
SS3
SS4
VH
VL
VL
VH
C
VL
β-domain
SS1
SS4
SS3
SS2
cleft
VH
α-domain
D
RMSF per Residue
Rigid 101D
Run1
Run2
Run3
RMSF (Angstroms)
Residue number
Flex 101D
Run1
Run2
Run3
RMSF (Angstroms)
Residue number

Fig. S1. Structure of HEL_{3X} self-lysozyme and molecular dynamics (MD) simulations of a flexible antigen variant. (A) Structural similarity between self-lysozyme HEL_{3X} (red cartoon and sticks, PDB entry 6p4d) and foreign DEL (blue cartoon and sticks, PDB entry 5v8g). Positions 21, 73 and 101 distinguish HEL_{3X} from wild-type HEL. Position 75 is the crucial surface difference between the HEL_{3X} and DEL lysozyme exploited by mutating Hy10 B cells. Positions of amino acid differences are indicated by stick representation of side chains. **(B-C)** Relative positions of the four lysozyme disulfides. Lysozyme contains four disulfide bonds (SS1-SS4), distributed such that two reside in the α-domain, one in the β-domain, with the last, SS4, bridging between these two domains. Sulfur atoms of cysteine residues are shown as yellow spheres. **(D)** Root mean square fluctuation calculated for each residue of $Rigid_{R101D}$ and $Flex_{R101D}$ using three independent molecular dynamics simulations (1μs each).

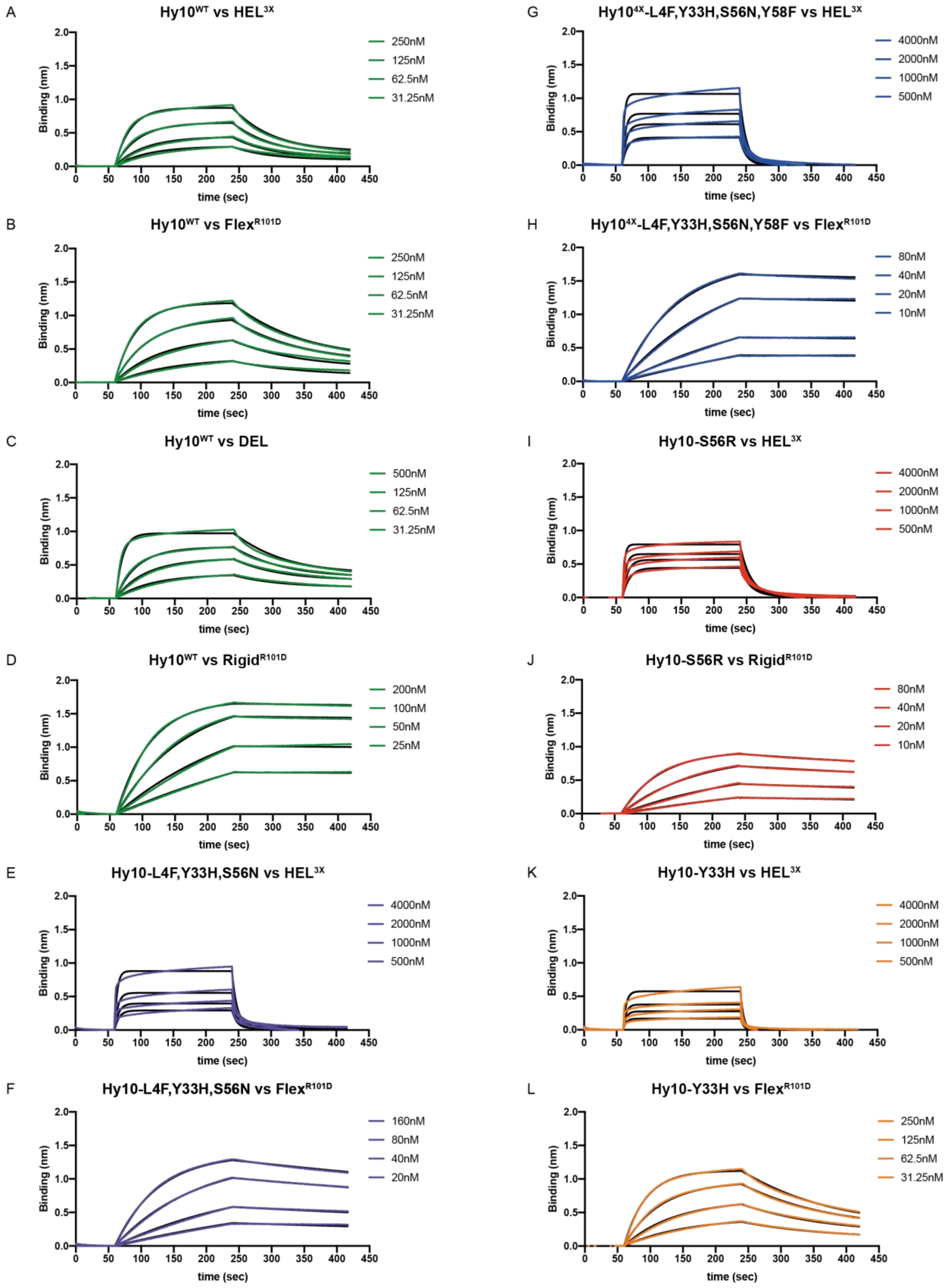
A
Hy10WT vs HEL3X
250nM
125nM
62.5nM
31.25nM
B
Hy10WT vs FlexR101D
250nM
125nM
62.5nM
31.25nM
C
Hy10WT vs DEL
500nM
125nM
62.5nM
31.25nM
D
Hy10WT vs RigidR101D
200nM
100nM
50nM
25nM
E
Hy10-L4F,Y33H,S56N vs HEL3X
4000nM
2000nM
1000nM
500nM
F
Hy10-L4F,Y33H,S56N vs FlexR101D
160nM
80nM
40nM
20nM
G
Hy104X-L4F,Y33H,S56N,Y58F vs HEL3X
4000nM
2000nM
1000nM
500nM
H
Hy104X-L4F,Y33H,S56N,Y58F vs FlexR101D
80nM
40nM
20nM
10nM
I
Hy10-S56R vs HEL3X
4000nM
2000nM
1000nM
500nM
J
Hy10-S56R vs RigidR101D
80nM
40nM
20nM
10nM
K
Hy10-Y33H vs HEL3X
4000nM
2000nM
1000nM
500nM
L
Hy10-Y33H vs FlexR101D
250nM
125nM
62.5nM
31.25nM
Binding (nm)
time (sec)

Fig. S2. Bio-layer interferometry. Experimental curves of association and disassociation of soluble monomeric protein antigens at the indicated concentrations binding to biotinylated Hy10 antibody Fab mutants immobilized onto streptavidin biosensors. Global fits are overlaid in black.

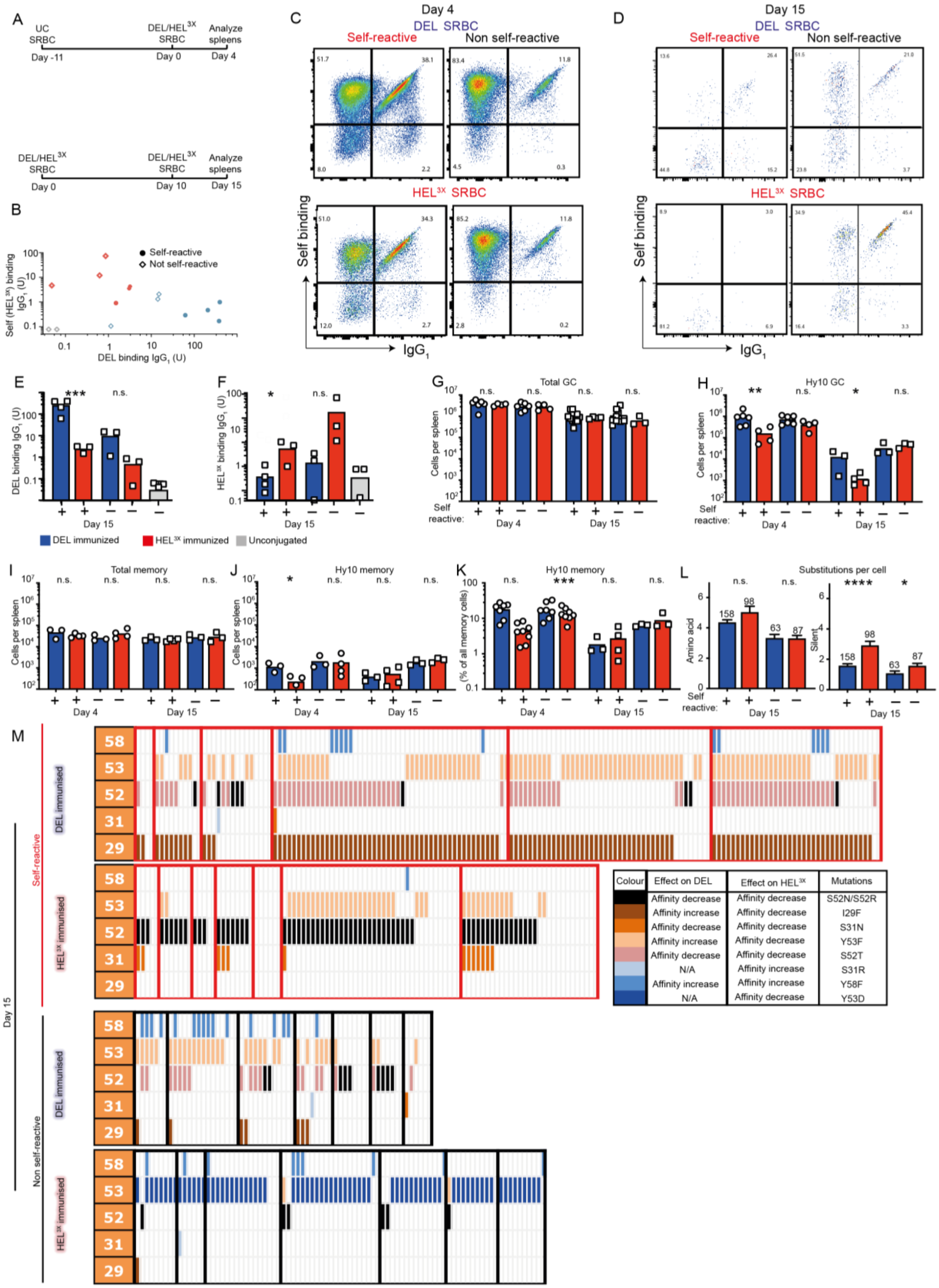
A
UC SRBC
DEL/HEL3X SRBC
Analyze spleens
Day -11
Day 0
Day 4
DEL/HEL3X SRBC
DEL/HEL3X SRBC
Analyze spleens
Day 0
Day 10
Day 15
B
Self-reactive
Not self-reactive
Self (HEL3X) binding IgG1 (U)
DEL binding IgG1 (U)
C
Day 4
DEL SRBC
Self-reactive
Non self-reactive
HEL3X SRBC
Self binding
IgG1
D
Day 15
DEL SRBC
Self-reactive
Non self-reactive
HEL3X SRBC
Self binding
IgG1
E
DEL binding IgG1 (U)
Day 15
DEL immunized
HEL3X immunized
Unconjugated
F
HEL3X binding IgG1 (U)
Day 15
G
Total GC
Cells per spleen
Self reactive:
Day 4
Day 15
H
Hy10 GC
Cells per spleen
Self reactive:
Day 4
Day 15
I
Total memory
Cells per spleen
Day 4
Day 15
J
Hy10 memory
Cells per spleen
Day 4
Day 15
K
Hy10 memory
(% of all memory cells)
Day 4
Day 15
L
Substitutions per cell
Amino acid
Silent
Self reactive:
Day 15
Day 15
M
Self-reactive
DEL immunised
HEL3X immunised
Non self-reactive
DEL immunised
HEL3X immunised
Day 15
58
53
52
31
29
Colour
Effect on DEL
Effect on HEL3X
Mutations
Affinity decrease | Affinity decrease | S52N/S52R
Affinity increase | Affinity decrease | I29F
Affinity decrease | Affinity decrease | S31N
Affinity increase | Affinity decrease | Y53F
Affinity decrease | Affinity decrease | S52T
N/A | Affinity increase | S31R
Affinity increase | Affinity increase | Y58F
N/A | Affinity decrease | Y53D

Fig S3. Autoantibody redemption requires a path for escape of self-reactivity. (A) Timing of chimera immunizations. (B) Total serum HEL_{3X} or DEL binding IgG_1 from chimeras harvested on day 15 after antigen exposure. (C-D) Representative flow cytometric analysis of binding to self (0.14uM HEL_{3X}) and cell surface IgG_1 of CD45.1+ GC B cells 4 (C) and 15 (D) days post immunization with the indicated antigens. (E) Total serum DEL binding IgG_1 from chimeras harvested on day 15 following antigen exposure. (F) Total serum HEL_{3X} binding IgG_1 from chimeras harvested on day 15 following antigen exposure. (G) Total GC cells (B220+Fas+CD38-) per spleen from individual mice at the indicated timepoints post antigen exposure. (H) Total SW_{HEL} GC cells (B220+Fas+CD38-CD45.1+CD45.2-) per spleen from individual mice at the indicated timepoints post antigen exposure. (I) Total IgG_1 memory cells (B220+Fas-CD38+IgG_1+) per spleen from individual mice at the indicated timepoints post antigen exposure. (J) Total SW_{HEL} IgG_1 memory cells (B220+Fas-CD38+IgG1+CD45.1+CD45.2-) per spleen from individual mice at the indicated timepoints post antigen exposure. (K) Percentage of SW_{HEL} cells amongst IgG_1 memory B-Cells from mice with or without self-HEL_{3X} immunized at the indicated timepoints after antigen exposure. (L) Average numbers of synonymous mutations and non-synonymous per Hy10 B GC cell at day 15. (M) Summary of mutations at H-chain I29, S31, S52, Y53, and Y58 in individual sorted Hy10 GC B cells. Color coding denotes the consequence of each mutation for self-affinity. Each column represents a single cell, each row denotes whether that cell has a mutation at the indicated amino acid position, and all the cells from a single mouse are grouped within red or black boxes. Data pooled from 2-3 independent experiments per timepoint with 1-4 mice per group. *P<0.05, **P<0.01, ***P<0.001,****P<0.001 by Student’s T test. Each data point represents a single mouse.

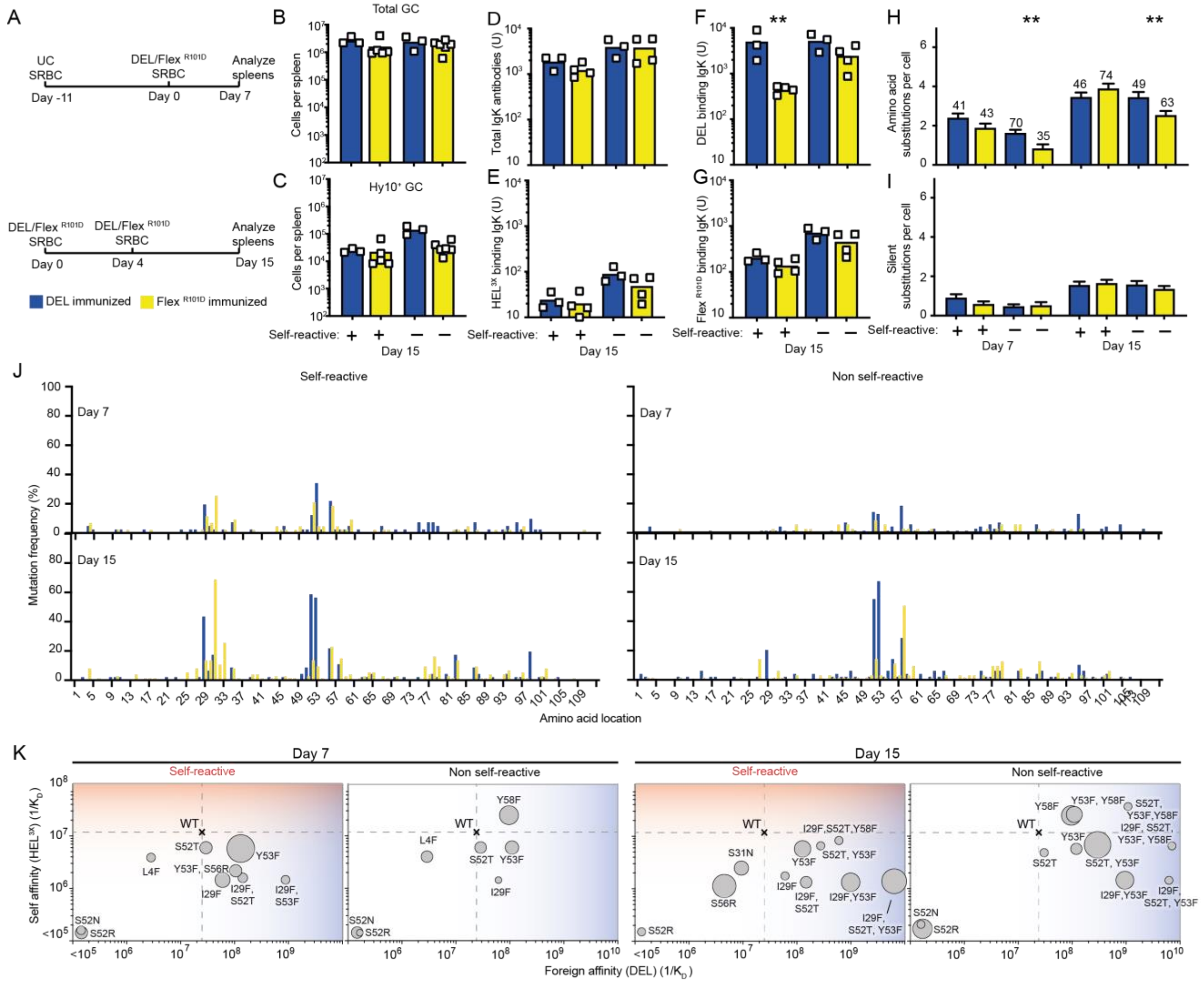

Fig. S4. Autoantibody redemption against a flexible antigen (I). (A) Timing of chimera immunizations. (B) Total GC cells (B220+Fas+CD38-) per spleen from individual mice at 15 days post antigen exposure. (C) Total SW$_{HEL}$ GC (B220+Fas+CD38-CD45.1+CD45.2-) per spleen from individual mice at 15 days post antigen exposure. (D) Total serum IgK from chimeras harvested on day 15 following antigen exposure. (E) Total HEL$_{3X}$ binding serum IgK from chimeras harvested on day 15 following antigen exposure. (F) Total DEL binding serum IgK from chimeras harvested on day 15 following antigen exposure. (G) Total Flex$_{R101D}$ binding serum IgK from chimeras harvested on day 15 following antigen exposure. (H) Average numbers of synonymous mutations per GC Hy10 B cell. (I) Average numbers of non-synonymous mutations per CD45.1$_{+}$ GC B cell. **P<0.01 Student's T test. Data points represent one mouse. (J) Percentage of CD45.1$_{+}$ GC B cells with substitutions at each H-chain amino acid. (K) Mutational trajectory of Hy10-expressing B cells towards self and foreign antigens following DEL immunization. Circles show the affinity of recurring mutant antibodies for self and foreign proteins, with area denoting the percentage of Hy10 B-cells with the indicated mutation. Data represents 2 independent experiments per timepoint with 1-2 mice per group.

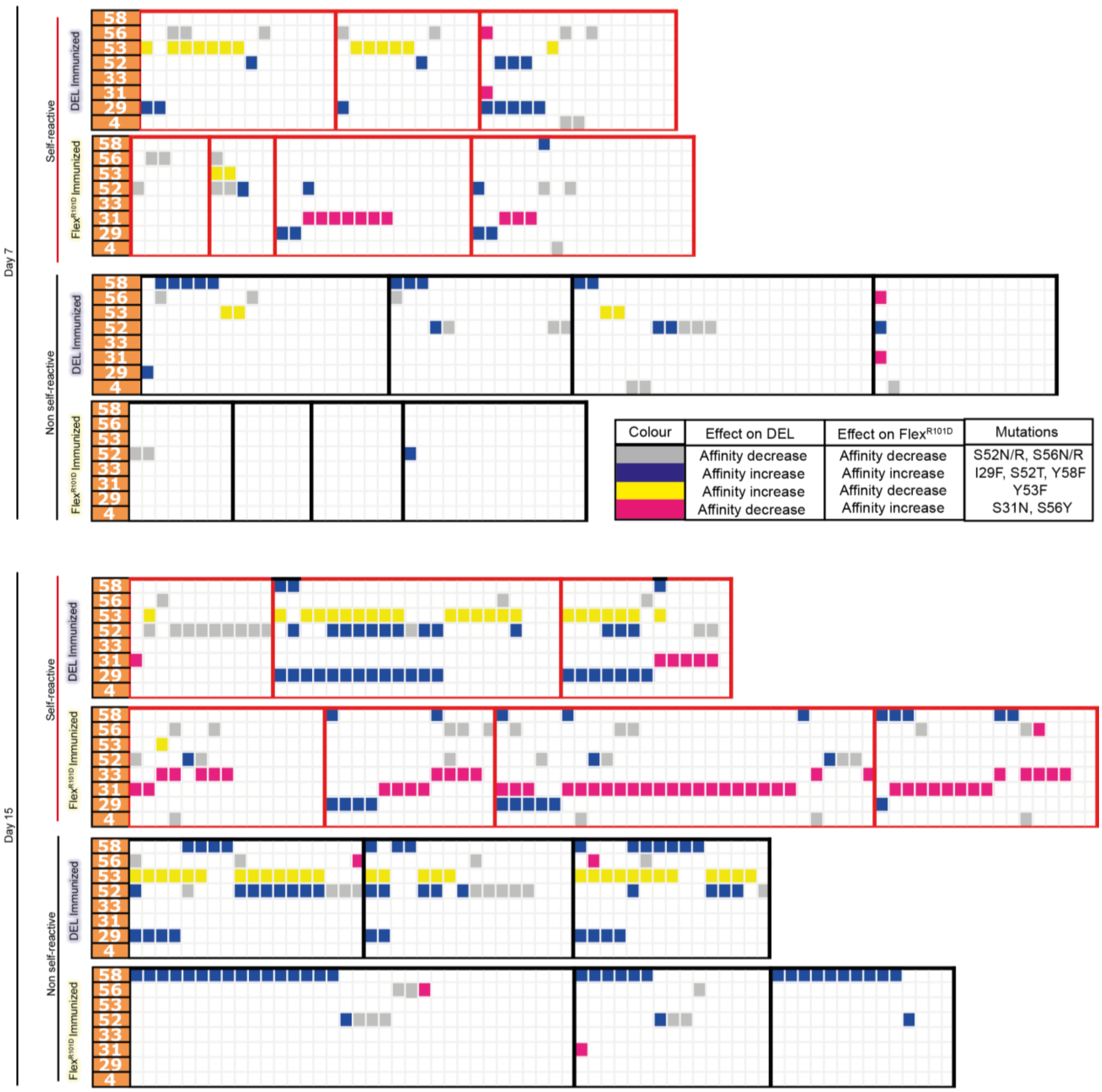

Fig. S5. Autoantibody redemption against a flexible antigen (II). Summary of mutations at H-chain L4, I29, S31, Y33 S52, Y53, S56 and Y58 in individual sorted CD45.1+ GC B cells. Color coding denotes the consequence of each mutation for DEL and Flex$_{R101D}$ affinity. Each column represents a single cell, each row denotes whether that cell has a mutation at the indicated amino acid position, and all the cells from a single mouse are grouped within red or black boxes. Data representative of 3 independent experiments with 3-4 mice per group.

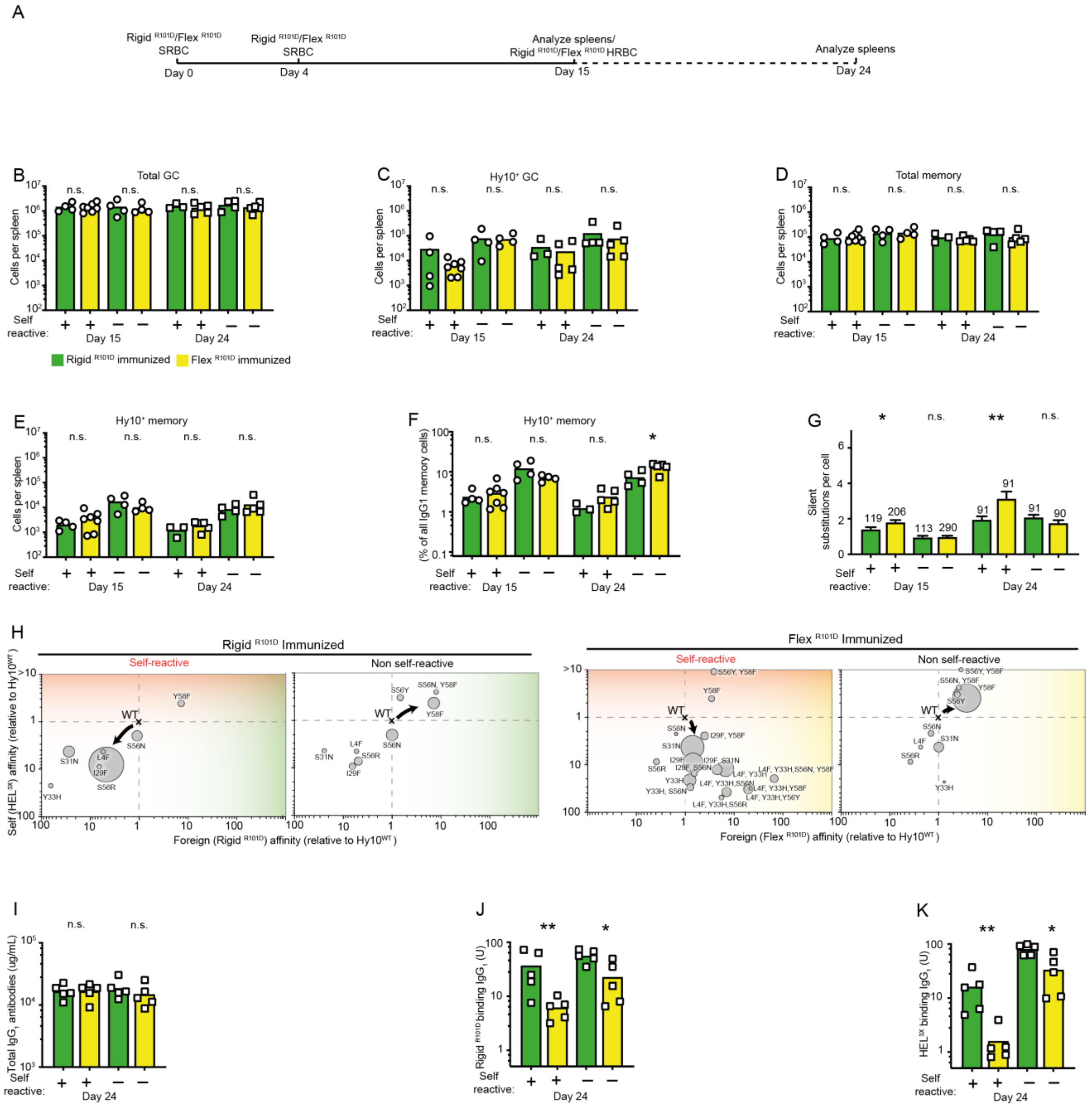

Fig. S6. Antigen flexibility enables B cells to explore diverse mutational trajectories leading to loss of self-reactivity and high affinity for foreign antigen (I). (A) Timing of chimera immunizations. (B) Total GC cells (B220+Fas+CD38-) per spleen from individual mice at the indicated timepoints post antigen exposure. (C) Total SW_{HEL} GC (B220+Fas+CD38-CD45.1+CD45.2-) per spleen from individual mice at 15 days post antigen exposure. (D) Total IgG_1 memory cells (B220+Fas-CD38+IgG_1+) per spleen from individual mice at the indicated timepoints post antigen exposure. (E) Total SW_{HEL} IgG_1 memory cells (B220+Fas-CD38+IgG_1+CD45.1+CD45.2-) per spleen from individual mice at the indicated timepoints post antigen exposure. (F) Percentage of SW_{HEL} cells amongst IgG_1 memory B-Cells from mice with or without self-HEL_{3X} immunized with $Flex_{R101D}$ or $Rigid_{R101D}$ at the indicated timepoints after antigen exposure. (G) Average number of non-synonymous mutations per $CD45.1_{+}$ GC B cell. (H) Mutational trajectory of Hy10-expressing GC B cells at day 15 relative to Hy10 founding affinity.

Circles show the affinity of recurring mutant antibodies for self and foreign proteins, with area denoting the percentage of $CD45.1^+$ GC B-cells with the indicated mutation. (I) Total serum IgG_1 from chimeras harvested on day 15 following antigen exposure. (J) Total $Rigid_{R101D}$ binding serum IgG_1 from chimeras harvested on day 15 following antigen exposure. (K) Total HEL_{3X} binding serum IgG_1 from chimeras harvested on day 15 following antigen exposure. *P<0.05,**P<0.01 Student's T test. Data points represent one mouse. Data representative of 2 independent experiments per timepoint with 2-3 mice per group.

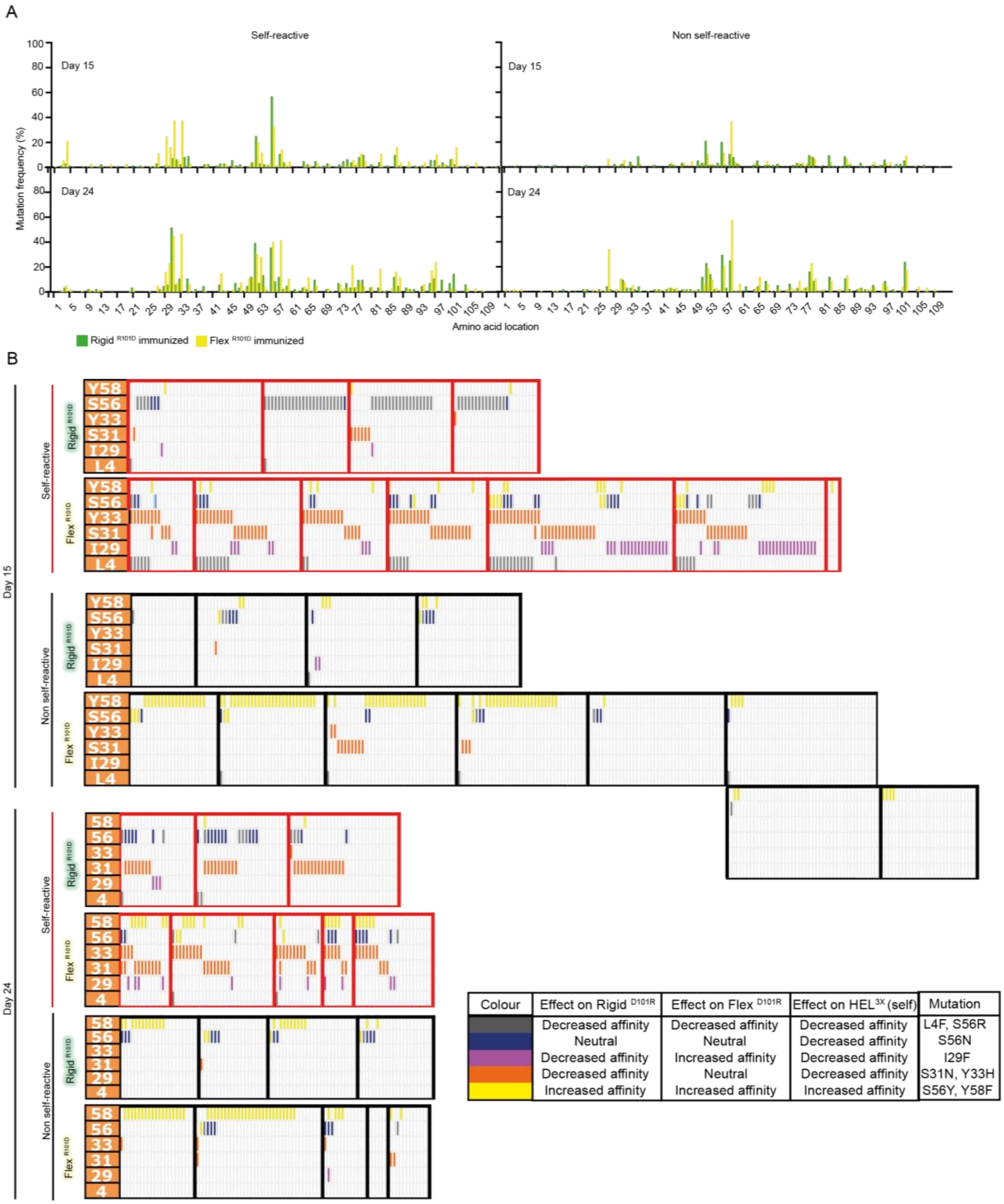

Fig. S7. Antigen flexibility enables B cells to explore diverse mutational trajectories leading to loss of self-reactivity and high affinity for foreign antigen (II). (A) The percentage of CD45.1+ GC B cells with substitutions at each H-chain amino acid at the indicated timepoints post antigen exposure. (B) Summary of mutations at H-chain positions L4, I29, S31, Y33, S56 and Y58 in individual sorted CD45.1+ GC B cells. Each column represents a single cell, each row denotes whether that cell has a mutation at the indicated amino acid position, and all the cells from a single

mouse are grouped within red or black boxes. Data representative of 2 independent experiments per timepoint with 2-3 mice per group.

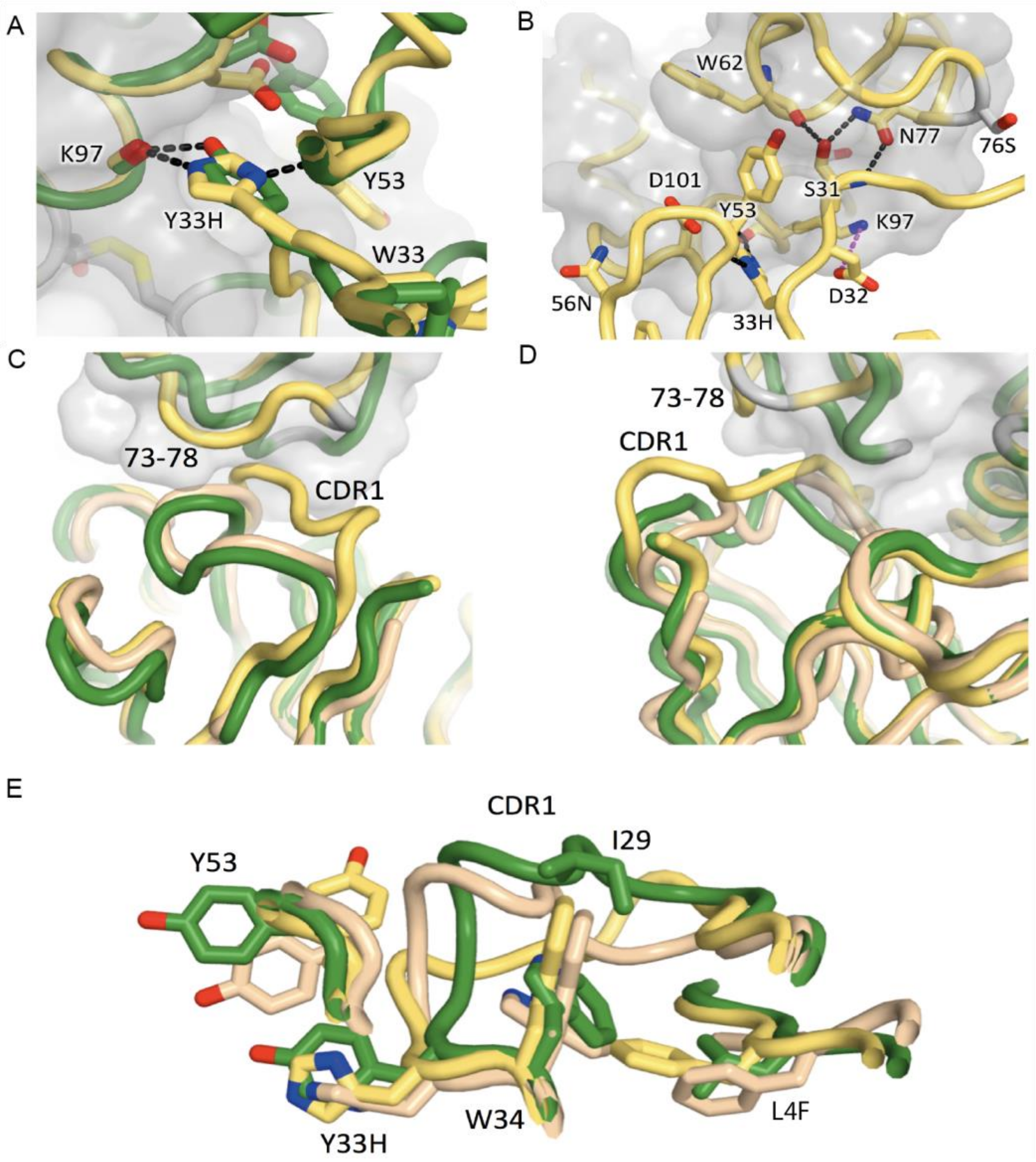

Fig. S8. Structural details of the Hy10$_{4X}$-Flex$_{R101D}$ complex (yellow), comparison with Hy10-Rigid$_{R101D}$ (green) and the unliganded Hy10$_{4X}$ structure (tan). (A) The Hy10$_{4X}$-Flex$_{R101D}$ complex is stabilized by Y33H forming two additional hydrogen bonds (black dashed lines), thereby linking antibody (Y53) and antigen (K97) backbones. (B) Additional hydrogen bonds connect Hy10$_{4X}$ and Flex$_{R101D}$: (I) through the repositioned antibody residue S31 contacting W62 main-chain and N77 side-chain (antigen), (II) through an additional salt bridge (magenta dashed line) connecting D32 of Hy10$_{4X}$ and K97 of Flex$_{R101D}$. (C-E) Structure of unliganded Hy10$_{4X}$ (tan) superposed onto the structures of Hy10-Rigid$_{R101D}$ (green) and Hy10$_{4X}$-Flex$_{R101D}$ (yellow). Two perspectives are shown (C and D), with the 73-78 loop of lysozyme and CDRH1 of Hy10 highlighted. (E) Details of Hy10 interface focusing on framework mutation L4F and conformations of W34 and I29 of CDRH1. In the unliganded Hy10$_{4X}$ structure W34 can be modelled as a mix of two conformers, reflecting an overall intermediary conformation, with the positioning of L4F more reminiscent of the Hy10-Rigid$_{R101D}$ interaction and only partial rearrangement of antibody CDRH1 conformation in comparison to Hy10$_{4x}$-Flex$_{R101D}$.

Data collection statistics				
**Protein**	**HEL_{3X}**	**$Hy10_{4X}$**	**Flex $_{R101D}$ $Hy10_{4X}$**	**Rigid $_{R101D}$ Hy10**
Wavelength	0.9794	0.9537	0.9537	0.9537
Spacegroup	P 4_3 2_1 2	P 1 2 1	P 1 2_1 1	P 1 2_1 1
Unit cell dimensions: a, b, c (Å); α, β, γ, (°)	77.55, 77.55, 37.77; *90.00, 90.00, 90.00*	68.56, 40.16, 93.65; *90.00, 107.57, 90.00*	66.40, 65.72, 137.12; *90.00, 98.48, 90.00*	63.62, 55.24, 74.23; *90.00, 90.46, 90.00*
Resolution range	1.05-38.77 (1.05-1.07)	1.85-46.47 (1.85-1.89)	1.90-37.25 (1.90-1.93)	2.20-48.50 (2.20-2.27)
Total reflections	1294996 (61767)	275598 (15523)	620458 (27676)	178665 (14648)
Unique reflections	54091 (2610)	42030 (2577)	92222 (4546)	26223 (2181)
Completeness	100 (100)	100 (100)	99.9 (99.9)	99.4 (97.0)
Multiplicity	23.9 (23.7)	6.6 (6.0)	6.7 (6.1)	6.8 (6.7)
Average (I/σ(I))	20.6 (4.5)	16.8 (2.0)	12.5 (2.2)	11.9 (2.7)
Mean half set correlation, $CC_{1/2}$	0.998 (0.947)	0.999 (0.778)	0.998 (0.693)	0.998 (0.899)
Rmeas (all I+ and I-)	0.086 (0.637)	0.056 (0.947)	0.106 (0.927)	0.102 (0.703)
Rpim (all I+ and I-)	0.018 (0.132)	0.022 (0.386)	0.041 (0.373)	0.039 (0.267)
Wilson B ($Å^2$)	9.4	37.5	25.0	32.8
**Refinement and model statistics**				
R_{work}/R_{free}	0.122/0.145	0.188/0.223	0.203/0.244	0.229/0.292
Molecules/asu	1*HEL	1*Fab	2*HEL-Fab	1*HEL-Fab
Atoms protein	1175	3221	8289	4196
B average protein ($Å^2$)	13.2	42.6	34.0	41.63
Atoms water	169	139	457	62
B average water ($Å^2$)	26.5	43.0	33.0	34.0
RMSD bond lengths (Å)	0.0102	0.0119	0.0093	0.0074
RMSD bond angles (°)	1.72	1.78	1.62	1.56
Ramachandran Outliers (%)	0.00	0.48	0.38	0.37
Ramachandran Favored (%)	97.6	96.86	96.25	97.43
**PDB entry**	**6p4d**	**6p4c**	**6p4b**	**6p4a**

Table S1. Diffraction data and model refinement statistics. Values in parentheses represent values for the highest resolution shell.

HEL$_{3X}$ (self) affinities					
**Hy10 variant**	**K_D [M]**	**k_a [$M^{-1}s^{-1}$]**	**k_d [s^{-1}]**	**K_A [M^{-1}]**	**Affinity**
WT founder	9.35x10^{-8}	1.25x10^{5}	1.17x10^{-2}	1.07x10^{7}	94 nM
L4F	2.49x10^{-7}	1.33x10^{5}	3.30x10^{-2}	4.02x10^{6}	249 nM
L4F/S31N	2.43x10^{-7}	8.99x10^{4}	2.19x10^{-2}	4.11x10^{6}	243 nM
L4F/Y33H	1.15x10^{-6}	1.65x10^{5}	1.89x10^{-1}	8.71x10^{5}	1.15 µM
L4F/Y33H/S56N	3.70x10^{-6}	3.25x10^{4}	1.20x10^{-1}	2.70x10^{5}	3.7 µM
L4F/Y33H/S56N/Y58F	1.85x10^{-6}	5.31x10^{4}	9.83x10^{-2}	5.41x10^{5}	1.8 µM
L4F/Y33H/S56R	4.44x10^{-6}	2.20x10^{4}	9.78x10^{-2}	2.25x10^{5}	4.4 µM
L4F/Y33H/S56Y	3.18x10^{-6}	3.17x10^{4}	1.01x10^{-1}	3.15x10^{5}	3.2 µM
L4F/Y33H/Y58F	2.33x10^{-6}	4.43x10^{4}	1.03x10^{-1}	4.29x10^{5}	2.3 µM
I29F	7.65x10^{-7}	1.04x10^{5}	7.93x10^{-2}	1.31x10^{6}	765 nM
I29F/S31N	1.24x10^{-6}	6.31x10^{4}	7.79x10^{-2}	8.10x10^{5}	1.2 µM
I29F/S52T	6.10x10^{-7}	6.98x10^{4}	4.26x10^{-2}	1.64x10^{6}	610 nM
I29F/S52T/Y53F	8.07x10^{-7}	8.64x10^{4}	6.97x10^{-2}	1.24x10^{6}	807 nM
I29F/S52T/Y53F/Y58F	1.48x10^{-7}	1.07x10^{5}	1.58x10^{-2}	6.76x10^{6}	148 nM
I29F/S52T/Y58F	1.13x10^{-7}	1.14x10^{5}	1.28x10^{-2}	8.88x10^{6}	113 nM
I29F/Y53F	7.98x10^{-7}	9.85x10^{4}	7.86x10^{-2}	1.25x10^{6}	798 nM
I29F/Y58F	2.21x10^{-7}	1.14x10^{5}	2.51x10^{-2}	4.53x10^{6}	221 nM
I29F/S56N	1.37x10^{-6}	3.63x10^{4}	4.96x10^{-2}	7.33x10^{5}	1.4 µM
S31N	3.88x10^{-7}	1.16x10^{5}	4.49x10^{-2}	2.58x10^{6}	388 nM
S31N/S52R	Does not detectably bind at 5 µM				N/A
S31N/S52R/Y53F	Does not detectably bind at 5 µM				N/A
S31N/S56N	3.62x10^{-7}	9.54x10^{4}	3.46x10^{-2}	2.76x10^{6}	362 nM
S31N/Y58F	1.23x10^{-7}	1.23x10^{5}	1.50x10^{-2}	8.15x10^{6}	123 nM
Y33H	2.35x10^{-6}	7.75x10^{4}	1.82x10^{-1}	4.26x10^{5}	2.4 µM
Y33H/S56N	2.72x10^{-6}	5.49x10^{4}	1.49x10^{-1}	3.67x10^{5}	2.7 µM
Y33H/Y58F	2.31x10^{-6}	8.32x10^{4}	1.92x10^{-1}	4.34x10^{5}	2.3 µM
S52R	Does not detectably bind at 5 µM				N/A
S52R/Y53F	Does not detectably bind at 5 µM				N/A
S52T	1.67x10^{-7}	1.21x10^{5}	2.03x10^{-2}	5.98x10^{6}	167 nM
S52T/Y58F	5.14x10^{-8}	1.89x10^{5}	9.69x10^{-3}	1.95x10^{7}	51 nM
S52T/Y53F	1.52x10^{-7}	1.12x10^{5}	1.71x10^{-2}	6.58x10^{6}	152 nM
S52T/Y53F/Y58F	2.44x10^{-8}	2.08x10^{5}	5.07x10^{-3}	4.11x10^{7}	24 nM
Y53D	1.35x10^{-9}	1.08x10^{5}	1.46x10^{-4}	7.42x10^{8}	1.4 nM
Y53D/Y58F	2.33x10^{-10}	1.10x10^{5}	2.57x10^{-5}	4.29x10^{9}	233 pM
Y53F	1.64x10^{-7}	1.21x10^{5}	1.98x10^{-2}	6.11x10^{6}	164 nM
Y53F/S56R	4.63x10^{-7}	8.25x10^{4}	3.81x10^{-2}	2.16x10^{6}	463 nM
Y53F/Y58F	3.55x10^{-8}	9.55x10^{4}	3.39x10^{-2}	2.82x10^{7}	36 nM
S56N	1.91x10^{-7}	9.96x10^{4}	1.90x10^{-2}	5.23x10^{6}	191 nM
S56N/Y58F	2.23x10^{-8}	1.87x10^{5}	4.17x10^{-3}	4.49x10^{7}	22 nM
S56R	8.09x10^{-7}	7.31x10^{4}	5.92x10^{-2}	1.24x10^{6}	809 nM

S56Y	$3.13x10^{-8}$	$1.54x10^{5}$	$4.81x10^{-3}$	$3.19x10^{7}$	31 nM
S56Y/Y58F	$8.31x10^{-9}$	$1.79x10^{5}$	$1.49x10^{-3}$	$1.20x10^{8}$	8.3 nM
Y58F	$3.76x10^{-8}$	$1.93x10^{5}$	$7.24x10^{-3}$	$2.66x10^{7}$	38 nM

**DEL affinities**					
**Hy10 variant**	**K_D [M]**	**k_a [$M^{-1}s^{-1}$]**	**k_d [s^{-1}]**	**K_A [M^{-1}]**	**Affinity**
WT founder	$4.01x10^{-8}$	$1.85x10^{5}$	$7.40x10^{-3}$	$2.49x10^{7}$	40 nM
L4F	$3.08x10^{-7}$	$8.71x10^{4}$	$2.68x10^{-2}$	$3.25x10^{6}$	308 nM
L4F/Y33H	$1.72x10^{-7}$	$1.22x10^{5}$	$2.11x10^{-2}$	$5.80x10^{6}$	172 nM
I29F	$1.57x10^{-8}$	$1.76x10^{5}$	$2.76x10^{-3}$	$6.37x10^{7}$	16 nM
I29F/S52T	$5.79x10^{-9}$	$1.55x10^{5}$	$8.97x10^{-4}$	$1.73x10^{8}$	5.8 nM
I29F/S52T/Y53F	$1.64x10^{-10}$	$3.18x10^{5}$	$5.22x10^{-5}$	$6.10x10^{9}$	164 pM
I29F/S52T/Y53F/Y58F	$1.33x10^{-10}$	$2.10x10^{5}$	$2.79x10^{-5}$	$7.51x10^{9}$	133 pM
I29F/S52T/Y58F	$1.69x10^{-9}$	$1.91x10^{5}$	$3.22x10^{-4}$	$5.92x10^{8}$	1.7 nM
I29F/Y53F	$1.03x10^{-9}$	$1.47x10^{5}$	$1.52x10^{-4}$	$9.70x10^{8}$	1.0 nM
S31N	$1.04x10^{-7}$	$1.33x10^{5}$	$1.39x10^{-2}$	$9.60x10^{6}$	104 nM
Y33H	$5.94x10^{-7}$	$7.18x10^{4}$	$4.26x10^{-2}$	$1.68x10^{6}$	594 nM
S52R	Does not detectably bind at 5 µM				N/A
S52R/Y53F	Does not detectably bind at 5 µM				N/A
S52T	$3.35x10^{-8}$	$1.73x10^{5}$	$5.79x10^{-3}$	$2.98x10^{7}$	34 nM
S52T/Y58F	$2.62x10^{-9}$	$1.74x10^{5}$	$4.58x10^{-4}$	$3.80x10^{8}$	2.6 nM
S52T/Y53F	$3.56x10^{-9}$	$2.00x10^{5}$	$7.11x10^{-4}$	$2.81x10^{8}$	3.6 nM
S52T/Y53F/Y58F	$5.02x10^{-10}$	$2.54x10^{5}$	$1.27x10^{-4}$	$1.99x10^{9}$	502 pM
Y53F	$8.35x10^{-9}$	$2.18x10^{5}$	$1.82x10^{-3}$	$1.20x10^{8}$	8.4 nM
Y53F/S56R	$2.68x10^{-8}$	$1.67x10^{5}$	$4.49x10^{-3}$	$3.73x10^{7}$	27 nM
Y53F/Y58F	$8.85x10^{-9}$	$1.22x10^{5}$	$1.08x10^{-3}$	$1.13x10^{8}$	8.9 nM
S56R	$2.27x10^{-7}$	$1.31x10^{5}$	$2.96x10^{-2}$	$4.41x10^{6}$	227 nM
Y58F	$1.07x10^{-8}$	$2.63x10^{5}$	$2.81x10^{-3}$	$9.35x10^{7}$	11 nM

Rigid$_{R101D}$ affinities					
**Hy10 variant**	**K_D [M]**	**k_a [$M^{-1}s^{-1}$]**	**k_d [s^{-1}]**	**K_A [M^{-1}]**	**Affinity**
WT founder	$7.36x10^{-10}$	$9.54x10^{4}$	$7.02x10^{-5}$	$1.36x10^{9}$	736 pM
L4F	$3.94x10^{-9}$	$1.04x10^{5}$	$4.10x10^{-4}$	$2.54x10^{8}$	3.9 nM
L4F/Y33H	$1.14x10^{-8}$	$1.22x10^{5}$	$1.38x10^{-3}$	$8.77x10^{7}$	11 nM
L4F/Y33H/S56N	$3.07x10^{-9}$	$1.23x10^{5}$	$3.78x10^{-4}$	$3.26x10^{8}$	3.1 nM
L4F/Y33H/S56N/Y58F	$9.76x10^{-10}$	$1.52x10^{5}$	$1.48x10^{-4}$	$1.02x10^{9}$	976 pM
L4F/Y33H/S56R	$8.27x10^{-9}$	$1.43x10^{5}$	$1.19x10^{-3}$	$1.21x10^{8}$	8.3 nM
L4F/Y33H/S56Y	$1.58x10^{-9}$	$1.50x10^{5}$	$2.37x10^{-4}$	$6.33x10^{8}$	1.6 nM
L4F/Y33H/Y58F	$1.98x10^{-9}$	$1.15x10^{5}$	$2.27x10^{-4}$	$5.05x10^{8}$	2.0 nM
I29F	$4.77x10^{-9}$	$1.01x10^{5}$	$4.83x10^{-4}$	$2.10x10^{8}$	4.8 nM
I29F/S31N	$5.35x10^{-10}$	$1.56x10^{5}$	$8.35x10^{-5}$	$1.87x10^{9}$	535 pM
I29F/S56N	$5.76x10^{-9}$	$1.46x10^{5}$	$8.39x10^{-4}$	$1.74x10^{8}$	5.8 nM
I29F/Y58F	$1.27x10^{-9}$	$1.19x10^{5}$	$1.50x10^{-4}$	$7.87x10^{8}$	1.3 nM
S31N	$8.10x10^{-9}$	$7.72x10^{4}$	$6.26x10^{-4}$	$1.23x10^{8}$	8.1 nM
Y33H	$5.01x10^{-8}$	$9.36x10^{4}$	$4.69x10^{-3}$	$2.00x10^{7}$	50 nM
Y33H/S56N	$1.24x10^{-8}$	$1.68x10^{5}$	$2.08x10^{-3}$	$8.06x10^{7}$	12 nM
S56N	$8.13x10^{-10}$	$1.82x10^{5}$	$1.48x10^{-4}$	$1.23x10^{9}$	813 pM
S56N/Y58F	$9.54x10^{-11}$	$2.63x10^{5}$	$2.50x10^{-5}$	$1.05x10^{10}$	95 pM
S56R	$3.71x10^{-9}$	$2.04x10^{5}$	$7.56x10^{-4}$	$2.70x10^{8}$	3.7 nM
S56Y	$4.90x10^{-10}$	$1.71x10^{5}$	$8.36x10^{-5}$	$2.04x10^{9}$	490 pM
S56Y/Y58F	$4.59x10^{-10}$	$1.69x10^{5}$	$7.75x10^{-5}$	$2.18x10^{9}$	459 pM
Y58F	$1.02x10^{-10}$	$1.89x10^{5}$	$1.93x10^{-5}$	$9.80x10^{9}$	102 pM

Flex$_{R101D}$ affinities					
**Hy10 variant**	**K_D [M]**	**k_a [$M^{-1}s^{-1}$]**	**k_d [s^{-1}]**	**K_A [M^{-1}]**	**Affinity**
WT founder	$6.42x10^{-8}$	$9.91x10^{4}$	$6.36x10^{-3}$	$1.56x10^{7}$	64 nM
L4F	$1.50x10^{-7}$	$8.83x10^{4}$	$1.33x10^{-2}$	$6.65x10^{6}$	150 nM
L4F/S31N	$9.22x10^{-8}$	$1.66x10^{5}$	$1.53x10^{-2}$	$1.08x10^{7}$	92 nM
L4F/Y33H	$9.29x10^{-9}$	$9.06x10^{4}$	$8.42x10^{-4}$	$1.08x10^{8}$	9.3 nM
L4F/Y33H/S56N	$9.21x10^{-9}$	$9.18x10^{4}$	$8.45x10^{-4}$	$1.09x10^{8}$	9.2 nM
L4F/Y33H/S56N/Y58F	$9.58x10^{-10}$	$1.53x10^{5}$	$1.47x10^{-4}$	$1.04x10^{9}$	958 pM
L4F/Y33H/S56R	$1.42x10^{-8}$	$1.60x10^{5}$	$2.27x10^{-3}$	$7.05x10^{7}$	14 nM
L4F/Y33H/S56Y	$3.32x10^{-9}$	$1.58x10^{5}$	$5.24x10^{-4}$	$3.01x10^{8}$	3.3 nM
L4F/Y33H/Y58F	$2.94x10^{-9}$	$1.02x10^{5}$	$3.00x10^{-4}$	$3.40x10^{8}$	2.9 nM
I29F	$4.20x10^{-8}$	$1.18x10^{5}$	$4.93x10^{-3}$	$2.38x10^{7}$	42 nM
I29F/S31N	$1.47x10^{-8}$	$2.39x10^{5}$	$3.52x10^{-3}$	$6.79x10^{7}$	15 nM
I29F/S52T	$1.04x10^{-7}$	$8.18x10^{4}$	$8.49x10^{-3}$	$9.63x10^{6}$	104 nM
I29F/S52T/Y53F	$1.77x10^{-8}$	$1.33x10^{5}$	$2.35x10^{-3}$	$5.65x10^{7}$	18 nM
I29F/Y53F	$4.37x10^{-8}$	$1.03x10^{5}$	$4.50x10^{-3}$	$2.29x10^{7}$	44 nM
I29F/S56N	$4.13x10^{-8}$	$1.28x10^{5}$	$5.27x10^{-3}$	$2.42x10^{7}$	41 nM
I29F/Y58F	$2.71x10^{-8}$	$8.77x10^{4}$	$2.37x10^{-3}$	$3.70x10^{7}$	27 nM
S31N	$6.49x10^{-8}$	$1.18x10^{5}$	$7.67x10^{-3}$	$1.54x10^{7}$	65 nM
S31N/S56N	$5.97x10^{-8}$	$2.60x10^{5}$	$1.55x10^{-2}$	$1.68x10^{7}$	60 nM
S31N/Y58F	$2.31x10^{-8}$	$2.12x10^{5}$	$4.89x10^{-3}$	$4.33x10^{7}$	23 nM
Y33H	$5.29x10^{-8}$	$1.01x10^{5}$	$5.32x10^{-3}$	$1.89x10^{7}$	53 nM
Y33H/S56N	$4.67x10^{-8}$	$8.74x10^{4}$	$4.08x10^{-3}$	$2.14x10^{7}$	47 nM
Y33H/Y58F	$1.45x10^{-8}$	$2.11x10^{5}$	$3.06x10^{-3}$	$6.88x10^{7}$	15 nM
S52T	$8.37x10^{-8}$	$2.38x10^{5}$	$1.99x10^{-2}$	$1.19x10^{7}$	84 nM
S52T/Y58F	$3.46x10^{-8}$	$1.12x10^{5}$	$3.86x10^{-3}$	$2.89x10^{7}$	35 nM
S52T/Y53F	$4.30x10^{-8}$	$1.21x10^{5}$	$5.20x10^{-3}$	$2.32x10^{7}$	43 nM
S52T/Y53F/Y58F	$1.45x10^{-8}$	$1.77x10^{5}$	$2.58x10^{-3}$	$6.88x10^{7}$	15 nM
S56N	$8.38x10^{-8}$	$1.24x10^{5}$	$1.04x10^{-2}$	$1.19x10^{7}$	84 nM
S56N/Y58F	$2.59x10^{-8}$	$1.32x10^{5}$	$3.42x10^{-3}$	$3.86x10^{7}$	26 nM
S56R	$2.51x10^{-7}$	$7.81x10^{4}$	$1.96x10^{-2}$	$3.98x10^{6}$	251 nM
S56Y	$2.77x10^{-8}$	$1.45x10^{5}$	$4.01x10^{-3}$	$3.61x10^{7}$	28 nM
S56Y/Y58F	$1.64x10^{-8}$	$1.01x10^{5}$	$1.65x10^{-3}$	$6.12x10^{7}$	16 nM
Y58F	$1.72x10^{-8}$	$1.21x10^{5}$	$2.08x10^{-3}$	$5.83x10^{7}$	17 nM

Table S2. Affinity of lysozyme antigen variants.
Hy10 variants identified by single cell sequencing were expressed in a Fab format. Binding affinities of biotinylated Fab for soluble antigens (Self HEL$_{3X}$, DEL, Rigid$_{R101D}$ and Flex$_{R101D}$) were determined Biolayer Interferometry (BLI) as previously described (*14*).

Movie S1. Structural basis of autoantibody redemption against a flexible antigen. The conformational differences observed between $Rigid_{R101D}$ (green) and $Flex_{R101D}$ (yellow) are enabled by the disulfide bond disruption due to C76S and C94S mutations. The altered antigen conformation is stabilized by the rearrangement of the CDR1 region of the antibody variable heavy domain (VH).

Movie S2. Structural basis of autoantibody redemption against a flexible antigen. This movie shows the conformational differences in the antibody paratope CDRH1 between the complex of Hy10 with $Rigid_{R101D}$ (green) and $Hy10_{4X}$ bound to $Flex_{R101D}$ (yellow), promoted by the antibody H-chain mutations L4F and Y33H and rotation of the side chain of conserved antibody H-chain residue W34.
